# Comparative Analysis of Bone Remodeling Dynamics and Bone Vitality Following Standard Bone Augmentation Procedures: A Review

**DOI:** 10.3390/jfb17080398

**Published:** 2026-08-12

**Authors:** Nikola Gapińska, Marek Śmielecki, Kornel Krasny

**Affiliations:** 1 Doctoral School of Quantitative and Natural Sciences, Maria Curie-Skłodowska University, Weteranów 18, 20-038 Lublin, Poland; 2 Centrum Stomatologii Śmieleccy, Osiedle Władysława Łokietka 33, 62-200 Gniezno, Poland; 3 MEDICARE Stomatologia sp. z o.o., Mireckiego 13a/11, 05-300 Mińsk Mazowiecki, Poland; kornel.krasny@op.pl

**Keywords:** bone augmentation, dental biomaterials, bone remodeling, vital bone, allografts, biological functionalization, residual graft material, xenografts, alloplastic bone substitutes, histomorphometry

## Abstract

Bone augmentation procedures are widely used in implant dentistry to reconstruct deficient alveolar bone and support implant placement. However, outcomes are often evaluated mainly by defect filling, volumetric stability, or implant survival, which do not necessarily reflect the biological quality of regenerated tissue. This narrative review synthesizes human and supporting mechanistic evidence across autogenous, allogeneic, xenogeneic, and alloplastic grafts, including biologically functionalized constructs, with emphasis on bone remodeling dynamics and functional regeneration. Available evidence indicates that volumetric preservation alone should not be considered equivalent to complete bone regeneration and that remodeling is material-, product-, processing-, and indication-dependent. Augmented sites may contain newly formed vital bone together with persistent residual graft particles, forming a hybrid tissue whose significance depends on graft integration, vascularization, remodeling capacity, and the intended therapeutic objective. Persistent particles may influence the local immune microenvironment and, under unfavorable conditions, contribute to foreign body reactions or other complications, although such events appear uncommon and causal relationships remain uncertain. Biomaterial assessment should integrate quantitative, structural, and biological parameters, while residual graft persistence should be interpreted in an indication-specific manner rather than as evidence of treatment failure or inferior long-term performance in isolation.

## 1. Introduction

Bone augmentation procedures have become a fundamental component of modern implant dentistry and regenerative therapy, enabling the reconstruction of deficient bone structures necessary for functional and aesthetic rehabilitation [1]. The increasing demand for implant-based treatments, together with the high prevalence of bone loss resulting from tooth extraction, trauma, or disease [2,3,4], has contributed to the widespread use of bone grafting materials aimed at restoring the volume and structural integrity of the alveolar ridge [2,5]. In clinical practice, bone grafting is routinely performed in procedures such as ridge augmentation, sinus floor elevation, socket preservation, and periodontal surgery, where adequate bone volume is essential for predictable implant placement and long-term stability [6,7,8,9,10,11].

While small defects may heal spontaneously [12], larger defects typically require surgical intervention and the use of biomaterials to support bone regeneration [12,13,14,15]. In this context, graft materials serve as structural and biological scaffolds that guide cellular migration, vascular ingrowth, and new bone formation, thereby influencing not only the quantity but also the biological quality of the regenerated tissue [5,16,17,18].

A wide range of bone graft materials has been developed and can be broadly classified into autografts, allografts, xenografts, and alloplastic substitutes. These materials differ in their biological properties, particularly in terms of osteogenesis, osteoinduction, and osteoconduction, which are considered critical mechanisms for successful bone regeneration [5,12]. Among them, autogenous bone has traditionally served as a biological reference material because it provides viable osteogenic cells, osteoinductive signals, and an osteoconductive matrix [19,20]. Its clinical suitability, however, is indication- and donor-site-dependent, and its use is constrained by donor-site morbidity, limited availability, graft resorption, and increased surgical complexity [13,21,22]. Accordingly, autogenous bone should not be regarded as an unconditional gold standard for every augmentation procedure.

In response to these limitations, allogeneic, xenogeneic, and synthetic/alloplastic biomaterials have gained significant popularity due to their availability, avoidance of or reduction in donor-site morbidity, and indication-dependent handling and clinical performance [23,24]. In particular, deproteinized bovine bone mineral (DBBM) has become one of the most extensively used materials in implant dentistry, largely due to its structural similarity to human bone and its capacity to provide long-term volumetric stability [11,25]. However, this stability is often associated with slow or incomplete resorption, resulting in the long-term persistence of residual graft particles within the augmented site [5,25].

Although preservation of graft volume has traditionally been considered an important indicator of clinical success, increasing evidence suggests that volumetric stability alone does not necessarily reflect the biological quality of regenerated bone [5]. Bone regeneration is a complex process involving not only the formation of new tissue, but also its vascularization, remodeling, and functional adaptation over time. When biological replacement is the principal objective, graft materials should undergo controlled resorption and be progressively replaced by vital, well-organized host bone capable of physiological turnover; in indications prioritizing space or contour maintenance, controlled persistence of a well-integrated scaffold may also be clinically appropriate [5,12,26]. In clinical reality, however, many commonly used biomaterials, particularly slowly resorbing xenografts, may result in the formation of a composite structure in which newly formed bone coexists with persistent residual graft particles [27,28,29,30,31,32].

This phenomenon raises important questions regarding the long-term biological performance of augmented bone and its ability to provide optimal support for implant stability and function. Despite the extensive use of bone graft materials in clinical practice, there remains no clear consensus regarding the extent to which different biomaterials are replaced by native bone or how this process affects the biological quality of the regenerated tissue. Moreover, the concept of vital bone is not consistently defined or systematically incorporated as an outcome parameter, as many studies primarily focus on volumetric, radiographic, or histomorphometric outcomes rather than the functional properties of regenerated tissue [5,33].

In the present review, the term “bone remodeling dynamics” refers to the temporal sequence and balance of biological events that determine the composition and functionality of an augmented site. These events include vascular ingrowth and graft integration, the formation and maturation of new vital bone, the resorption, remodeling, or persistence of graft material, and the subsequent capacity of the regenerated tissue to participate in physiological turnover and functional adaptation.

Unlike previous reviews that focus primarily on implant survival, dimensional changes, individual augmentation procedures, or selected categories of grafting materials, the present review integrates histological, histomorphometric, radiological, and biological evidence across the principal graft categories. Its added value lies in examining the relationship between vital bone formation, residual graft material, volumetric stability, and remodeling potential, thereby distinguishing structural preservation of the augmented site from functional biological regeneration.

Contemporary augmentation strategies increasingly seek to modify the biological response to a scaffold through the addition or controlled delivery of signaling molecules, bioactive macromolecules, or cellular components. In the present review, biological functionalization refers to the combination of a graft or scaffold with such an exogenous biological adjunct to enhance cell recruitment, proliferation, or differentiation, angiogenesis, and new bone formation [34,35,36]. Because these interventions may alter the amount and temporal progression of vital bone formation, vascular ingrowth, graft integration, and subsequent turnover, they are considered modifiers of biomaterial performance rather than intrinsic properties of the underlying graft [37,38].

Therefore, the aim of this review is to critically evaluate bone remodeling dynamics and the biological quality of tissue formed following augmentation procedures, with particular emphasis on the persistence of residual graft material and its potential clinical implications. This review specifically examines whether regenerated tissue corresponds to the structural and functional properties of native bone or instead represents a hybrid structure composed of newly formed bone and residual biomaterial, whose biological and mechanical characteristics may differ from those of native bone.

## 2. Literature Search and Study Selection

This narrative review was developed using an iterative literature search conducted in PubMed between April and June 2026 and updated during subsequent manuscript revisions through August 2026. The search aimed to identify evidence relevant to the biological quality and remodeling of bone formed following oral and maxillofacial augmentation procedures. Because the review covered several interconnected clinical, histological, and biological aspects, topic-specific combinations of keywords were used rather than a single fixed search string.

The search terms included combinations of keywords related to augmentation procedures, such as “bone augmentation”, “alveolar ridge augmentation”, “guided bone regeneration”, “ridge preservation”, “socket preservation”, “sinus floor augmentation”, and “sinus floor elevation”. These terms were combined with keywords describing the principal graft categories and materials, including “autogenous bone”, “autograft”, “allogeneic bone”, “allograft”, “FDBA”, “DFDBA”, “xenograft”, “deproteinized bovine bone mineral”, “alloplastic bone substitute”, “hydroxyapatite”, “beta-tricalcium phosphate”, “biphasic calcium phosphate”, and “bioactive glass”.

Additional search terms addressed outcomes relevant to the scope of the review, including “bone remodeling”, “new bone formation”, “vital bone”, “functional bone regeneration”, “vascularization”, “graft resorption”, “residual graft material”, “histology”, “histomorphometry”, “osseointegration”, “foreign body reaction”, and “bone graft complications”. Targeted searches were also performed for biologically functionalized materials and regenerative additives, including “BMP-2”, “BMP-7”, “PDGF”, “hyaluronic acid”, and “bone marrow aspirate concentrate”.

Priority was given to peer-reviewed English-language publications published between 2015 and 2026. Earlier studies were included when they represented landmark publications, provided long-term observations, or were directly relevant to the biological mechanisms and clinical outcomes discussed in the review. Eligible publications included human clinical studies, randomized and non-randomized comparative studies, histological and histomorphometric investigations, cohort studies, case series, systematic reviews, meta-analyses, and relevant narrative reviews. Selected experimental and mechanistic studies were included where necessary to explain biological processes that could not be adequately addressed using clinical evidence alone. Case reports were considered primarily in relation to uncommon complications and foreign body reactions, whereas case series were additionally included when they provided relevant human histological or longer-term clinical outcome data not available from higher-level evidence.

Publications were excluded when their titles or abstracts indicated that they did not concern oral or maxillofacial bone augmentation or did not report outcomes relevant to new bone formation, graft remodeling or resorption, residual biomaterial, volumetric stability, vascularization, tissue vitality, or biological integration. Potentially relevant publications were subsequently evaluated on the basis of their full text. The reference lists of relevant original articles and reviews were also manually screened to identify additional publications.

For consistency, the evidence discussed throughout this review was interpreted across five interrelated domains: (1) the amount and temporal progression of new vital bone formation, (2) vascular ingrowth, (3) graft integration with the surrounding host bone, (4) graft resorption, remodeling, or persistence, and (5) the capacity of the regenerated tissue for subsequent physiological turnover and functional adaptation. Not all included studies evaluated each of these domains; therefore, conclusions were based on the outcomes reported in the available evidence.

Recent human histological and histomorphometric evidence, direct comparisons between grafting materials, and studies with longer follow-up periods were prioritized whenever available. Systematic reviews and meta-analyses were used to characterize the broader evidence base, whereas individual primary studies were included to provide specific clinical, histological, and quantitative findings. Owing to the narrative character of this review and the heterogeneity of the included evidence, no formal risk-of-bias assessment or quantitative meta-analysis was performed.

The restriction of the search to PubMed and the narrative, topic-driven selection of publications may have resulted in the omission of some relevant studies and should be considered a limitation of the present review.

## 3. Biomaterials Used in Bone Augmentation: An Overview

**Figure 1:**
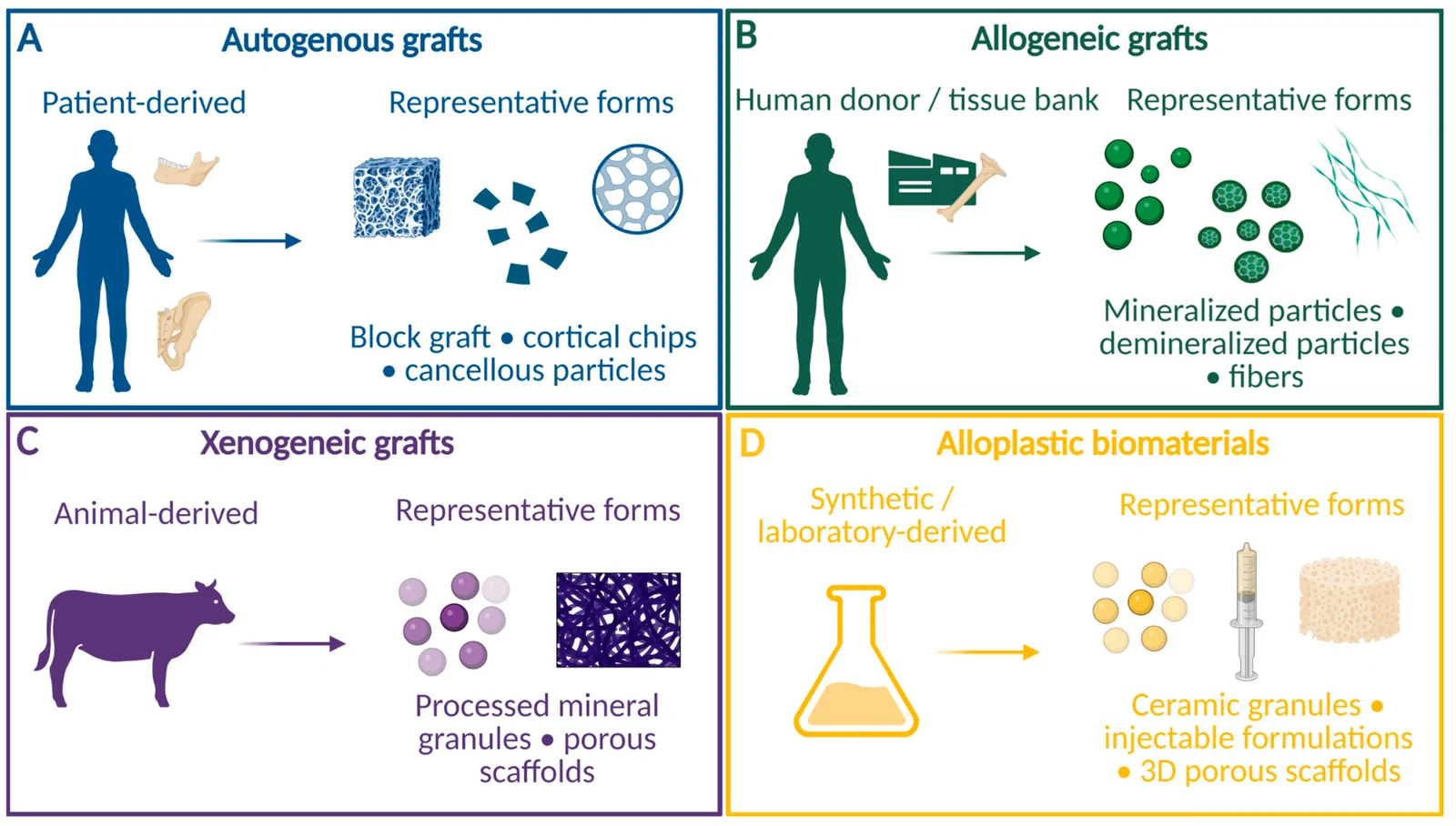
Representative origins and physical forms of the principal bone graft material categories used in dental bone augmentation. (**A**) Autogenous grafts are patient-derived and may be used as blocks, cortical chips, or cancellous particles. (**B**) Allogeneic grafts originate from human donors and are provided through tissue banks as mineralized or demineralized particles and fibers. (**C**) Xenogeneic grafts are animal-derived and are commonly processed into mineral granules or porous scaffolds. (**D**) Alloplastic biomaterials are synthetically manufactured and include ceramic granules, injectable formulations, and three-dimensional porous scaffolds. Created in BioRender. Gapińska, N. (2026) https://BioRender.com/mejutyp (accessed on 7 August 2026).

An overview of the principal sources and representative physical forms of autogenous, allogeneic, xenogeneic, and alloplastic graft materials is provided in Figure 1. Their general biological properties, advantages, and limitations are subsequently compared in Table 1.

### 3.1. Autogenous Bone Grafts

Autogenous bone grafts have traditionally served as a biological reference in bone augmentation because they combine viable osteogenic cells, endogenous osteoinductive signals, and an osteoconductive mineralized matrix [12,19,20,21,39]. Their biological and clinical performance nevertheless depends on graft architecture, donor site, recipient-site conditions, defect morphology, and the objective of treatment. Therefore, they cannot be regarded as an unconditional gold standard across all augmentation procedures, including sinus floor augmentation, in which volume maintenance and avoidance of an additional donor site may substantially influence material selection [40,41,42]. Nevertheless, autogenous bone remains an important option in selected cases requiring substantial biological activity or extensive reconstruction, provided that donor-site burden and the desired dimensional outcome are taken into account [39,40,43].

The biological advantage of autogenous grafts is primarily attributed to their ability to support bone regeneration through all three fundamental mechanisms: osteogenesis, osteoinduction, and osteoconduction [12,19,20,21,39]. The presence of viable osteogenic cells and bioactive growth factors enables direct participation in new bone formation, while the graft matrix provides a natural scaffold for tissue integration [12,21,39]. In addition, autografts are characterized by excellent biocompatibility, minimal immunogenic risk, and no donor-derived disease-transmission risk, which further supports their clinical effectiveness [19,20].

These properties make autogenous bone particularly valuable when a high level of biological activity is required, including selected large or complex defects and extensive ridge reconstructions [39,40,43]. However, the indication should be individualized according to the amount and architecture of available donor bone, the donor-site morbidity expected for a given harvesting location, the anatomical characteristics of the recipient site, and the need for long-term volume maintenance.

Despite their biological advantages, autografts are associated with several important clinical limitations. These include donor-site morbidity, limited availability of graft material, increased surgical time, and the need for an additional surgical procedure, which may result in postoperative pain, infection, scarring, and prolonged hospitalization. In addition, systemic factors such as advanced age, metabolic disorders including osteoporosis and diabetes, or impaired healing capacity may negatively affect regenerative outcomes [13,21,22,44,45,46,47].

Across the remodeling domains considered in this review, autogenous grafts generally show comparatively rapid integration, active new bone formation, and progressive remodeling by host tissue [19,20,40,48]. These processes may support the formation of biologically active bone capable of subsequent physiological turnover. At the same time, relatively rapid graft turnover may be accompanied by dimensional reduction during healing, and the magnitude of this change depends on graft type, donor site, surgical technique, and local mechanical conditions [41,42,49]. Thus, favorable biological replacement does not necessarily ensure optimal preservation of the augmented contour.

Overall, autogenous bone is treated in this review as a biological reference material rather than a universally superior graft. Its osteogenic and osteoinductive activity should be considered together with donor-site morbidity, availability, graft architecture, indication-dependent resorption, and dimensional requirements. Accordingly, material selection should be based on the clinical objective rather than on a fixed hierarchy.

### 3.2. Allogeneic Bone Grafts

Allogeneic bone grafts constitute a heterogeneous group of human donor-derived materials that eliminate the need for a second surgical donor site [5,39]. They include mineralized and demineralized preparations, cortical and cancellous matrices, and blocks, particles, or fibers, among which freeze-dried bone allograft (FDBA) and demineralized freeze-dried bone allograft (DFDBA) are frequently used [50,51,52]. Their biological behavior cannot be generalized across the entire category because it is influenced by donor-related factors, tissue-bank processing, the extent of demineralization, graft architecture and particle size, and whether the product is aseptically processed or subjected to terminal sterilization.

After processing, allografts generally do not contain viable donor osteogenic cells and therefore act primarily as osteoconductive matrices that support host-cell migration, vascular ingrowth, and new bone deposition. DFDBA may additionally exhibit osteoinductive activity because demineralization can expose bone morphogenetic proteins and other matrix-associated signals; however, the magnitude of this activity varies among donors, tissue banks, products, and processing protocols [11,50,51,52,53]. Consequently, older results obtained with one DFDBA preparation should not be extrapolated uncritically to all currently available allogeneic materials.

Donor screening and processing are necessary to support microbial safety and product standardization, but procedures such as irradiation, aggressive chemical treatment, or terminal sterilization may modify matrix structure, mechanical properties, and biological activity [50,51,52,53]. Contemporary aseptic procurement and processing protocols aim to preserve relevant matrix characteristics while maintaining microbial safety. Residual immunological or transmission risks should be acknowledged, but interpreted in relation to donor screening, processing, and regulatory quality control rather than presented as a uniform property of allografts [50,51,52,53].

Clinical selection of an allogeneic graft should be indication- and product-specific. Allografts can provide a useful balance between reduced surgical morbidity, availability, structural support, and biological remodeling, but the expected outcome depends on the particular preparation and cannot be inferred solely from its human origin or from the broad designation FDBA or DFDBA [20,21].

Across the remodeling domains considered in this review, allogeneic grafts may support vascular ingrowth, integration with host bone, and gradual replacement by newly formed vital bone, while residual graft particles may persist to a variable extent [48,53,54,55]. The rate and completeness of remodeling differ among mineralized and demineralized materials and among blocks, particles, and fibers. Recent human histological evidence indicates that selected modern aseptically processed DFDBA preparations can yield substantial vital bone formation and favorable graft turnover after ridge preservation; in a randomized clinical trial, DFDBA used alone produced more vital bone than combinations containing xenograft, while a fiber formulation left less residual allograft than particulate DFDBA [56]. These findings reinforce that processing and physical form are central determinants of allograft behavior.

Overall, allogeneic grafts should not be described simply as an intermediate category with uniformly moderate regenerative potential. They represent a diverse family of materials whose capacity for new vital bone formation, integration, graft resorption, and subsequent physiological remodeling is strongly product- and processing-dependent. Their clinical interpretation therefore requires consideration of the specific material, treatment indication, healing period, and histological outcome measured.

### 3.3. Xenogeneic Bone Grafts

Xenogeneic bone grafts are widely used in implant dentistry and are derived from non-human sources, most commonly bovine, although porcine-, equine-, and marine-derived materials are also available. They undergo processing intended to remove cellular and organic components, reduce antigenicity, and achieve a reproducible mineral scaffold [57,58,59,60]. As with allografts, xenografts should not be treated as a biologically uniform category because source species, processing temperature, purification, crystallinity, porosity, and particle characteristics influence their interaction with host tissue.

Bovine-derived materials are extensively used because of their long clinical history, porous mineral architecture, handling characteristics, and ability to support osteoconductive bone formation and maintenance of the augmented contour [21,61,62]. Their pore structure and interconnectivity can facilitate cellular migration, vascular ingrowth, and deposition of new bone along the graft surface [63,64,65,66]. These properties explain their value as space-maintaining scaffolds, particularly in indications in which dimensional stability is a major treatment objective.

Commercial xenografts differ in source, processing method, particle size, and physicochemical properties, and these differences may affect vascular infiltration, graft integration, resorption kinetics, residual particle persistence, and local mechanical behavior [67,68,69,70,71,72,73,74]. In particular, processing temperature, degree of purification, porosity, and hydroxyapatite crystallinity may shift the balance between long-term structural persistence and biological replacement by host bone [25]. Therefore, conclusions derived from one bovine or other xenogeneic product should not automatically be generalized to the entire material class.

From a biological perspective, xenografts primarily provide osteoconduction: they create a scaffold for host-cell migration, vascular ingrowth, and new bone deposition, but generally do not contain viable osteogenic cells and show little intrinsic osteoinductive activity [5,12]. Their regenerative outcome consequently depends strongly on recipient-site vascularity, the amount and quality of surrounding bone, defect morphology, membrane stability, and healing time.

A principal advantage of many xenogeneic materials is long-term volume maintenance, which is associated with structural stability and slow or incomplete graft resorption [5,11,25]. This behavior can be clinically desirable for space maintenance and contour preservation, but it represents only one outcome domain. Volumetric stability does not by itself establish the amount or maturation of new vital bone, the degree of vascularization and integration, or the capacity of the regenerated tissue for subsequent physiological turnover [12,75].

The healed site may therefore contain newly formed bone in direct contact with residual xenogeneic particles. Such a composite architecture is not synonymous with treatment failure: residual particles may remain well integrated and coexist with stable implants and favorable clinical outcomes. Their significance depends on the proportion and distribution of residual material, the amount and maturity of vital bone, local inflammatory findings, the intended clinical objective, and the duration of follow-up [12,75,76].

Potential limitations include slow biological turnover, dependence on host regenerative capacity, and patient-specific ethical or religious concerns related to animal origin. Modern purification and sterilization protocols are designed to minimize immunogenicity and pathogen transmission, and the remaining infectious risk is considered theoretical and very low; nevertheless, manufacturing quality and transparent product characterization remain relevant considerations [5,11,21,77].

In summary, xenogeneic grafts are clinically established osteoconductive scaffolds that can provide effective space maintenance, integration, and predictable dimensional support. Their generally slow graft turnover means that biological replacement may remain incomplete, but the presence of residual particles should be interpreted together with vital bone formation, vascular and structural integration, clinical stability, and the treatment objective rather than as an adverse outcome in isolation.

### 3.4. Alloplastic (Synthetic) Biomaterials

Alloplastic biomaterials represent synthetic bone substitutes that play an increasingly important role in modern implant dentistry and periodontology. In contrast to biological materials such as autografts, allografts, and xenografts, alloplasts are artificially manufactured from inorganic or organic components, allowing precise control over their chemical composition, structure, and physicochemical properties. This enables the design of materials tailored to specific clinical requirements and regenerative objectives [5].

Among synthetic grafts, calcium phosphate-based ceramics are the most widely used, including HA, β-tricalcium phosphate (β-TCP), and biphasic calcium phosphate (BCP), all of which closely resemble the mineral phase of human bone [5]. In addition, other materials, such as bioactive glass, calcium sulfate, biodegradable polymers, and advanced composite scaffolds, are also employed, typically in the form of granules, blocks, or porous three-dimensional constructs [12,78].

The development of synthetic biomaterials has been driven by the increasing demand for bone reconstruction materials and the limited availability of autogenous and allogeneic grafts [79]. As a result, new generations of biomaterials are being intensively developed, including bioactive ceramics and advanced composite systems designed to improve both biological performance and mechanical stability [12]. Within this group, calcium phosphate-based materials, particularly HA, play a central role. Because of its close similarity to the mineral phase of bone and excellent biocompatibility, HA is among the most extensively studied and widely used biomaterials in bone regeneration [80,81,82].

The primary mechanism by which alloplastic biomaterials support bone regeneration is osteoconduction [83]. These materials act as physical scaffolds that maintain the defect space and allow for host-cell migration and vascular ingrowth, thereby facilitating new bone formation along their surface. Due to their porous or trabecular structure, many alloplasts create a favorable environment for tissue infiltration and vascularization [84]. However, most alloplastic materials lack living cells and intrinsic osteoinductive factors capable of directly initiating bone formation. Consequently, most of them do not actively induce osteogenesis but rely primarily on the regenerative potential of the surrounding host tissue. Because alloplastic materials generally rely on host tissue, the rate and extent of new bone formation are formulation-, defect-, and host-dependent. Their clinical performance cannot be inferred from the osteoconductive mechanism alone and is compared with other graft categories in Section 7. To enhance their regenerative performance, these materials are often combined with growth factors, cellular components, or other bioactive agents, which may improve osteoinductive signaling and promote more effective bone regeneration [85,86].

A key advantage of synthetic biomaterials is the ability to control their degradation and resorption kinetics by modifying their composition and microstructure. This allows for materials with different remodeling profiles to be designed according to specific clinical needs, balancing volume maintenance with gradual replacement by newly formed bone [5,12].

Alloplastic materials also offer important clinical advantages related to safety, availability, and handling [87,88,89]. Unlike autografts, they eliminate the need for donor-site harvesting, thereby reducing surgical invasiveness, patient morbidity, and the risk of complications such as pain, infection, or scarring. Furthermore, they exhibit good biocompatibility, ease of manipulation, moldability, and, in some cases, injectability, which facilitates adaptation to the defect and may reduce operative time [90,91]. Their high availability, resulting from scalable manufacturing, represents an additional advantage over biologically derived graft materials [12].

Alloplastic biomaterials constitute a heterogeneous group characterized by scalable availability, absence of donor-site harvesting, tunable composition and resorption kinetics, and generally limited intrinsic osteogenic and osteoinductive activity. Their clinical role depends on the specific formulation, treatment indication, host conditions, and the required balance between graft replacement and volume maintenance.

Taken together, the main bone graft categories differ not only in their origin and biological activity, but also in their remodeling behavior, residual material persistence, and capacity for volume maintenance. Their principal characteristics, advantages, and limitations are summarized in Table 1.

**Table 1:** Comparative characteristics of the main bone graft material categories used in augmentation procedures.

Material Category	Origin and Representative Forms	Biological Properties	Remodeling and Volume Maintenance	Key Advantages and Limitations
Autogenous bone grafts	Patient-derived bone [19,20]	Osteogenic, osteoinductive, and osteoconductive; biological activity depends on donor site, graft architecture, and handling [12,19,20,21,39]	Comparatively rapid integration and host-bone replacement; remodeling and dimensional reduction are donor-site-, graft-, and indication-dependent [19,20,40,41,42,48,49]	Advantages: – viable osteogenic cells and endogenous biological signals – biological reference material – strong potential for vital bone formation [19,20]
				Limitations: – donor-site morbidity and limited availability – additional surgical procedure – possible volume reduction; suitability depends on indication [13,21,22,41,42,44,45,46,47,49]
Allogeneic bone grafts	Human donor bone; mineralized or demineralized; cortical/cancellous blocks, particles, or fibers, including FDBA and DFDBA [50,51,52]	Primarily osteoconductive; DFDBA may exhibit processing- and product-dependent osteoinductive activity; no viable donor osteogenic cells [11,50,51,52,53]	Highly processing- and formulation-dependent; remodeling and residual material vary among products [48,53,54,55]. Selected modern aseptically processed DFDBA formulations may support substantial vital bone formation and favorable graft turnover [56]	Advantages: – avoids donor-site morbidity – broad availability and multiple forms [5,20,21,39] – selected preparations may support substantial vital bone formation and remodeling [56]
				Limitations: – donor-, tissue-bank-, processing-, and product-dependent properties – variable osteoinductive activity and resorption – residual immunological or transmission risks require interpretation in the context of screening and processing [50,51,52,53]
Xenogeneic bone grafts	Animal-derived, mainly bovine, porcine, equine, or marine materials [57,58,59,60]	Predominantly osteoconductive; supports host-cell and vascular ingrowth but lacks viable osteogenic cells and has minimal intrinsic osteoinductive activity [5,12]	Usually slow or incomplete graft resorption with high volume maintenance; residual particles may remain integrated with vital bone and should be interpreted in clinical context [5,11,12,25,75,76]	Advantages: – effective space and contour maintenance – high availability and extensive clinical evidence – predictable osteoconductive integration in appropriate indications [21,25,62]
				Limitations: – slow material turnover and persistent residual particles – biological outcome depends strongly on host and site conditions – animal-origin considerations; residual material is not itself evidence of failure [5,11,12,25,75,76]
Alloplastic biomaterials	Synthetic materials, including HA, β-TCP, BCP, bioactive glass, calcium sulfate, polymers, and composite scaffolds [5,12,78]	Mainly osteoconductive; generally lack viable cells and intrinsic osteoinductive factors [83,84,85,86]	Material-dependent degradation; resorption kinetics and volume maintenance can be modified through material design [5,12]	Advantages: – high and scalable availability – no donor-site morbidity – customizable composition and resorption kinetics [5,12,87,88,89,90,91]
				Limitations: – low intrinsic biological activity – no viable osteogenic cells – regeneration depends mainly on host tissue [83,84,85,86]

Note: Biologically functionalized constructs are not presented as a separate graft category because their performance depends jointly on the properties of the base scaffold and the incorporated biological adjunct; they should therefore be interpreted as distinct modified interventions. Abbreviations: BCP, biphasic calcium phosphate; DFDBA, demineralized freeze-dried bone allograft; FDBA, freeze-dried bone allograft; HA, hydroxyapatite; β-TCP, beta-tricalcium phosphate.

## 4. Concept of an Ideal Biomaterial

Despite significant advances in materials used for bone augmentation, currently available biomaterials still have limitations and may not fully reproduce the biological, structural, and functional properties of native bone [88]. Consequently, the concept of an “ideal biomaterial” has been proposed as a reference framework for evaluating the clinical performance and biological effectiveness of various bone substitutes. This concept extends beyond the intrinsic properties of the material itself and encompasses its interaction with host tissues, its capacity for biological integration, and its long-term influence on the quality of the regenerated bone. In this context, the assessment of biomaterials should not be limited to short-term clinical outcomes, such as defect filling or volume preservation, but should also consider their ability to participate in bone remodeling processes [92].

An ideal biomaterial should exhibit excellent biocompatibility, allowing for integration with host tissues without eliciting adverse immune responses or chronic inflammation [92]. This includes not only the absence of cytotoxicity but also the ability to support a physiological healing response and promote tissue regeneration [93]. Of particular importance is the material’s capacity to facilitate key regenerative mechanisms, including osteoconduction and, when biologically functionalized, osteoinductive or osteogenic activity, thereby contributing to new bone formation [92]. Contemporary approaches also emphasize the critical role of material–cell interactions, including the influence of surface properties on cell adhesion, proliferation, and differentiation, which directly affect the overall regenerative outcome [94].

For biomaterials intended to undergo biological replacement, an important characteristic is controlled resorption accompanied by progressive formation of host bone [92]. This process should be coordinated with the rate of new tissue formation to ensure both initial structural stability and long-term biological integration [95,96]. A mismatch between degradation behavior and the therapeutic objective, including resorption that is too rapid to preserve space or persistence where replacement is required, may lead to suboptimal regenerative outcomes [97]. In indications prioritizing biological replacement, a major objective is therefore to obtain tissue that not only fills the defect but also resembles native bone in structure, vascularization, and remodeling capacity [34].

Increasing attention is being paid to the quality of regenerated bone, particularly its ability to adapt and remodel in response to functional loading. Bone remodeling is a continuous process governed by the balance between osteoclastic resorption and osteoblastic bone formation, leading to structural renewal and reorganization of bone tissue. Under physiological conditions, this process maintains bone homeostasis, supports adaptation to mechanical stimuli, and enables repair of microdamage [98]. In the context of bone augmentation, the ability of regenerated tissue to participate in remodeling is a key feature of functional bone regeneration [5,12,26]. Accordingly, regenerated tissue containing persistent residual biomaterial should be assessed in relation to host-bone formation and maturity, graft integration, the therapeutic objective, and long-term clinical performance rather than interpreted unfavorably on the basis of persistence alone [99].

Another critical criterion of an ideal biomaterial is its microarchitectural design, including porosity, pore size, and pore interconnectivity, which are essential for enabling cellular migration, vascular ingrowth, and the proper progression of regenerative processes [92]. These structural features directly influence nutrient diffusion, oxygen supply, and waste removal, all of which are fundamental for cell survival and tissue formation. In particular, an interconnected porous network facilitates angiogenesis, which is a prerequisite for sustained bone regeneration and subsequent remodeling [100]. Moreover, microstructural characteristics influence the spatial organization of newly formed bone and its integration with the surrounding host environment.

In addition to biological considerations, the biomaterial must exhibit appropriate mechanical properties, ensuring sufficient structural stability of the augmented site while allowing the transfer of functional loads. The balance between mechanical strength and porosity is therefore particularly important, as excessive density may hinder biological integration, whereas insufficient stability may compromise the regenerative outcome [101].

From a clinical perspective, the ideal biomaterial should be easy to handle, readily available in sufficient quantities, and associated with a high level of safety [92,93]. This includes a minimal risk of disease transmission, low immunogenic potential, and consistent clinical performance [102]. Practical handling characteristics are also highly relevant, including ease of manipulation, adaptability to defect morphology, and the ability to conform to irregular anatomical structures [12]. In addition, minimally invasive application techniques are increasingly favored, as they reduce surgical trauma, patient morbidity, and postoperative complications. Standardization and reproducibility of material properties further contribute to predictable clinical outcomes in modern implantology and regenerative procedures [12].

In clinical practice, a fundamental trade-off is often observed between volumetric stability and biological remodeling. Materials that provide long-term volume maintenance frequently exhibit limited resorption and reduced participation in physiological bone turnover [5,25]. Conversely, rapidly degrading materials may facilitate replacement by host bone but may not provide sufficient structural support during early healing [41,42,49,103,104]. This highlights the need to evaluate biomaterials not only in terms of volume preservation, but also with regard to their involvement in remodeling processes [92].

As a consequence, contemporary research should focus on the development of biomaterials capable of achieving a balance between structural stability and biological activity. This includes the design of materials with tunable resorption kinetics, optimized microarchitecture, and enhanced bioactivity. In parallel, significant attention is being directed toward tissue engineering strategies aimed at improving the regenerative potential of biomaterials through biological functionalization. Approaches such as the incorporation of growth factors, cells, or bioactive molecules seek to enhance osteogenic signaling, promote vascularization, and ultimately improve both the quantity and quality of newly formed bone [85,86]. These developments reflect a shift toward more biologically driven regenerative concepts, in which the goal is not only defect filling but the restoration of functional bone tissue capable of long-term remodeling and adaptation.

### Biological Functionalization of Bone Graft Materials

Biological functionalization extends the concept of an ideal biomaterial beyond passive scaffold properties. By combining an osteoconductive matrix with growth factors, bioactive molecules, or cellular concentrates, functionalization aims to enhance the host response while the scaffold maintains space and provides a surface for tissue ingrowth. Its effect is nevertheless determined by the identity and dose of the adjunct, release kinetics, carrier affinity, defect vascularity, and host conditions; consequently, outcomes obtained with a functionalized construct should not be attributed to the base material alone [34,35,36,92].

Among the most extensively investigated osteoinductive signals are bone morphogenetic proteins, particularly BMP-2 and BMP-7. These members of the transforming growth factor-beta superfamily promote osteogenic commitment and differentiation of progenitor cells. Recombinant human BMP-2 (rhBMP-2) has been evaluated clinically in alveolar socket preservation and ridge or sinus augmentation, with evidence of enhanced bone formation in selected carrier-based protocols [105]. The overall evidence remains heterogeneous, and conventional comparators frequently yield similar dimensional or clinical outcomes [37,38]. The regenerative effect of rhBMP-2 is strongly dose-, carrier-, and retention-dependent; poorly controlled or supraphysiological exposure may produce an excessive local tissue response or bone formation outside the intended compartment. rhBMP-2 should therefore be interpreted as a potent, indication-specific adjunct whose benefit depends on the delivery strategy rather than as a universal substitute for an osteoconductive scaffold [34,35,36].

BMP-7 has related osteogenic potential, but its evidence base in oral augmentation is substantially less mature.An earlier systematic assessment found that evidence for localized alveolar ridge augmentation was predominantly preclinical, andthe recent dental literature continues to describe BMP-7 as promising while emphasizing the need for controlled human trials and optimized delivery [106,107]. BMP-2 and BMP-7 should therefore not be treated as clinically interchangeable.

Platelet-derived growth factor, particularly recombinant human PDGF-BB (rhPDGF-BB), acts primarily through chemotactic and mitogenic signaling rather than direct osteoinduction. It recruits and expands mesenchymal and other reparative cell populations and may support vascularized wound healing when combined with an appropriate graft or scaffold. Experimental ridge-augmentation data demonstrate that its effect depends on the carrier construct [82], while a systematic review of 63 human studies reported favorable clinical outcomes with rhPDGF-BB combined with allografts or xenografts in guided bone regeneration and alveolar ridge preservation [108]. Nevertheless, acceleration of early cellular events does not by itself establish complete graft replacement or long-term physiological remodeling, and the evidence remains indication- and material-dependent [37,38,108].

Hyaluronic acid is an extracellular-matrix glycosaminoglycan used as a hydrated carrier and biological adjunct. Its viscoelastic and hygroscopic properties may improve clot stabilization, handling, cell migration, and local inflammatory or angiogenic signaling, but it should not be described as intrinsically osteoinductive. A 2024 systematic review and meta-analysis identified only three eligible randomized trials and found no statistically significant improvement in new bone formation or reduction in residual graft particles when hyaluronic acid was added to graft materials [109]. Current evidence therefore supports describing hyaluronic acid as a potentially useful adjunct whose effect depends on formulation, cross-linking, carrier material, and indication, rather than as a proven enhancer of bone remodeling.

Cellular concentrates represent another form of functionalization. Bone marrow aspirate concentrate (BMAC) provides a heterogeneous mixture of progenitor and hematopoietic cells together with soluble paracrine mediators, but its composition varies with aspiration, processing, and patient factors. In the comparative study by Sauerbier et al., bovine mineral combined with BMAC did not produce more new bone than the autogenous-bone combination during early healing and was associated with substantial residual bovine mineral [110]. This example illustrates that adding a biological component does not automatically improve each remodeling domain.

Accordingly, the present review includes biologically functionalized constructs when they provide relevant evidence on bone quality or remodeling, but interprets them separately from otherwise comparable unmodified grafts. Their evaluation should address the same five domains used throughout this review: new vital bone over time, vascular ingrowth, graft integration with host bone, graft resorption or persistence, and the capacity of the regenerated tissue for subsequent physiological turnover. Dimensional preservation or early bone fill alone is insufficient to establish a durable biological benefit. The comparative clinical evidence for these adjuncts is evaluated separately in Section 7.4.

## 5. Bone Remodeling Outcomes Following Augmentation Procedures

### 5.1. Concept of Vital Bone and Functional Bone Regeneration

In the field of bone regeneration, the term vital bone is widely used; however, it lacks a clear and universally accepted definition. In most studies, it is applied descriptively to refer to bone tissue exhibiting features of biological viability, such as the presence of living cells, vascularization, and the ability to participate in healing and remodeling processes [111,112]. Bone itself is recognized as a living and dynamic tissue that fulfills multiple physiological functions, including structural support, organ protection, mineral storage, and hematopoietic and endocrine roles [112,113,114].

From both physiological and pathological perspectives, bone vitality is commonly interpreted through markers of biological activity, including bleeding, inflammatory response, initiation of repair processes, and regenerative capacity [115]. Histological and histomorphometric analyses frequently distinguish between vital, necrotic, and regenerating bone areas; however, these classifications are functional rather than based on strictly standardized terminology [116]. Consequently, vital bone should be understood as a spectrum of biological characteristics rather than a strictly defined category.

Importantly, “newly formed bone” and “vital bone” should not be treated as interchangeable terms. Newly formed bone refers to tissue identified as having developed during healing, whereas vital bone denotes viable mineralized bone identified histologically and may include tissue at different stages of maturation. Because individual studies apply these labels differently, their terminology should be retained as reported and interpreted together with the assessment method and healing interval [117,118,119,120].

In the context of augmentation procedures, this ambiguity becomes particularly relevant. A key question is whether bone formed following the application of biomaterials truly retains the capacity for long-term adaptation and remodeling. In many studies, regenerative outcomes are assessed primarily on the basis of macroscopic defect filling or structural parameters, although these measures do not necessarily reflect the biological status of the newly formed tissue [121,122].

Another important limitation is the lack of clearly defined criteria for distinguishing fully vital tissue from structures that are only partially remodeled [116]. In clinical and experimental settings, simplified quantitative parameters are commonly used, including the percentage of newly formed bone, the amount of residual graft material, and the bone-to-biomaterial ratio within the defect area [123]. These parameters are typically assessed using histological and histomorphometric analyses, as well as imaging techniques such as micro-computed tomography (micro-CT) or conventional computed tomography (CT), which provide information on tissue composition, three-dimensional structure, and bone density [117,124,125]. Although these methods offer valuable insights into defect filling and structural organization, they do not fully capture cellular metabolic activity, vascularization, or the actual involvement of the tissue in physiological remodeling [126,127]. As a result, favorable quantitative or radiological outcomes do not necessarily correspond to biological functionality [105].

More advanced evaluation methods, including immunohistochemical analyses, assessment of osteoblastic and osteoclastic activity markers, and evaluation of angiogenesis, may provide a more detailed understanding of the biological processes occurring within regenerating bone [128,129]. Nevertheless, a universally accepted method for the comprehensive assessment of bone vitality is still lacking, and interpretation of regenerative outcomes requires consideration of both quantitative and functional aspects.

In this context, a functional approach to bone regeneration is gaining importance, in which the key criterion is the ability of the tissue to participate in remodeling processes and integrate with the physiological bone metabolism of the host [68,130]. Residual graft material represents an important variable in interpreting the biological functionality of regenerated bone and is discussed below in the context of graft persistence and, where replacement was expected, incomplete remodeling [118,131]. Recent human histological evidence provides a clinically relevant illustration of this distinction, showing that selected modern DFDBA preparations may yield substantial vital bone formation and markedly different residual-graft proportions despite comparable dimensional ridge changes [56]; these findings are discussed in detail in Section 7.2.

### 5.2. Residual Graft Persistence and Hybrid Bone Formation

Depending on the therapeutic objective, bone healing may follow different biologically and clinically acceptable pathways. When graft replacement is desired, the material may be gradually resorbed and replaced by newly formed bone that subsequently participates in physiological remodeling. In indications requiring durable space or contour maintenance, well-integrated residual particles may instead persist alongside newly formed bone. Numerous experimental and clinical studies indicate that graft material may remain within the tissue for extended periods, coexisting with newly formed bone [68].

The extent of this process is closely related to material characteristics, particularly its degradation profile, which depends on factors such as chemical composition, crystallinity, porosity, and particle size. For instance, β-TCP exhibits relatively high solubility and undergoes gradual resorption, facilitating its replacement by host bone [132,133]. In contrast, HA, due to its high crystallinity and chemical stability, degrades much more slowly [131,134]. This phenomenon is especially evident in xenografts such as DBBM, which may persist in the tissue for many years, maintaining its structure while participating only minimally in remodeling processes [29].

This outcome may be described as residual graft persistence or, when graft replacement was expected, as incomplete remodeling. The biomaterial may remain structurally integrated with newly formed bone, while its direct participation in physiological turnover remains limited [21,131,135,136].

In contrast to resorbable materials, which gradually degrade and may be replaced by host bone, materials with high structural stability persist within the tissue over long periods, providing primarily spatial support [5,68]. In certain clinical situations, however, prolonged material persistence may be desirable. This is particularly relevant in procedures requiring long-term space maintenance and contour stability, such as sinus floor elevation, ridge preservation, or reconstruction of defects prone to collapse [5,11]. In such cases, slowly resorbing materials may help preserve the augmented volume during the healing period, providing a stable scaffold for bone formation. However, this benefit should be interpreted in the context of limited material turnover, as long-term structural persistence does not necessarily correspond to complete biological remodeling [137].

From a biological perspective, key mechanisms governing bone regeneration include osteogenesis, osteoconduction, and osteoinduction, all of which influence the quality and nature of the newly formed tissue. Osteogenesis, associated with the presence of living cells capable of directly forming bone, is primarily observed in autogenous grafts and contributes to their high regenerative potential [12,136]. In contrast, most clinically used biomaterials rely predominantly on osteoconduction, a scaffold-dependent process in which bone growth occurs along the surface of the material [138,139]. Although osteoconduction enables cellular migration and vascularization, it does not actively initiate bone formation, potentially limiting the rate and extent of remodeling [140].

The physicochemical properties of biomaterials also play a critical role in shaping the regenerative process. Parameters such as porosity, pore size, and interconnectivity, as well as surface characteristics, influence the ability of the material to support angiogenesis and cellular infiltration [66,100,141]. In calcium phosphate-based materials, appropriate microarchitecture has been shown to promote osteoblast differentiation and integration with host bone [142,143]. Highly biocompatible and osteoconductive materials do not themselves provide viable osteogenic cells; their biological performance therefore depends on host-tissue recruitment, material microarchitecture and degradation behavior, and, where applicable, controlled biological functionalization [35,36,136,140,144,145,146].

Another important factor influencing regeneration is the interaction between the biomaterial and the immune system, described within the framework of osteoimmunology [68,147]. Bone healing is regulated by inflammatory and immune responses, particularly macrophage activity and polarization [148,149]. An imbalance between pro-inflammatory and pro-regenerative responses may delay healing and impair tissue integration [147,150,151].

To address these limitations, tissue engineering strategies have focused on improving the biological performance of biomaterials through functionalization with growth factors, cells, or bioactive molecules [34,35,144]. These approaches aim to enhance osteoinductive signaling and improve the quality of newly formed bone [35,36,145,146]. However, despite promising results, they do not necessarily predict the extent of scaffold replacement or persistence after functional loading, highlighting the complexity of biological processes involved in bone regeneration.

### 5.3. Biological Functionality of Regenerated Bone Tissue

The persistence of residual graft material and the formation of hybrid tissue raise important questions regarding the long-term biological and mechanical performance of regenerated bone [29,118]. Although newly formed bone may coexist in direct contact with biomaterial particles, such structural contact does not necessarily indicate complete biological remodeling. Instead, histologically observed bone–biomaterial contact may indicate osteoconductive integration, while residual particles may remain only partially resorbed within the regenerated tissue [29,152,153]. Therefore, it is important to distinguish between structural stability and biological functionality, as a regenerated site may maintain adequate volume and provide mechanical support while still containing non-resorbed components that do not participate in turnover in the same manner as native bone [68,131]. Such an outcome may be clinically acceptable, particularly when residual particles are well integrated and the therapeutic objective includes volume preservation, space maintenance, contour stability, or implant stabilization.

Residual graft persistence may also alter biomechanical behavior. Physiological bone continuously remodels in response to mechanical loading, thereby maintaining its structural and functional integrity [154,155]. From a mechanistic perspective, regions containing a substantial proportion of residual material may differ from native bone because biomaterial particles do not participate in the same dynamic turnover. This could contribute to local differences in mechanical properties and load distribution within regenerated bone and around an implant [156,157]. However, this remains a biological hypothesis and should not be equated with demonstrated clinical inferiority, because direct long-term correlations between residual material, adaptive capacity, and implant outcomes remain limited.

Residual graft particles may also influence the local biological microenvironment. Persistent biomaterial particles can interact with immune cells, including macrophages, and may affect the balance between pro-inflammatory and regenerative processes [147,148,158]. The nature and duration of this response are material- and context-dependent; limited degradability does not by itself establish harmful or chronic immune stimulation. In selected unfavorable situations, degradation products or persistent particles may promote prolonged macrophage activation and influence remodeling dynamics [151,159].

In addition to their mechanical and osteoconductive role, residual biomaterial particles may also be interpreted in the context of the foreign body response. After implantation, bone graft substitutes are recognized by host tissues as foreign materials and may initiate immune and inflammatory events involving protein adsorption, recruitment of inflammatory cells, macrophage activation, and, in some cases, the formation of foreign body giant cells [160,161]. Such a response does not necessarily represent pathological rejection, as controlled inflammation and macrophage-mediated degradation may participate in normal healing and material resorption. A chronic foreign body reaction is an uncommon, context-dependent outcome rather than an inherent consequence of particle persistence. When it occurs, the response may contribute to fibrotic tissue formation or impaired incorporation, whereas in other settings macrophage activity participates in normal material degradation [162].

Although such complications appear uncommon and are not systematically assessed in most clinical studies, human case reports and case series indicate that they may have clinical relevance. A symptomatic foreign body granuloma has been reported after the placement of hard tissue replacement material, a synthetic alloplastic bone substitute, in extraction sockets; histological analysis revealed numerous histiocytes and foreign body-type giant cells, and the patient’s symptoms improved after surgical removal of the grafting material [163]. In another case, guided bone regeneration (GBR) with anorganic bovine bone combined with autogenous bone and platelet-rich plasma resulted in the absence of regenerated hard tissue after 4 months; histology showed a fibrous connective tissue matrix containing anorganic bovine bone particles closely associated with multinucleated foreign body-type giant cells [164]. Case series involving bovine-derived xenografts have also described late complications, including graft migration, encapsulation or displacement of particles, chronic inflammation, oroantral communication, sinus infections, and foreign body reactions, suggesting that intact non-degraded xenograft particles may contribute to long-term clinical complications in selected cases [8,75,165]. In addition, a delayed granulomatous foreign body reaction has been described after lateral sinus augmentation with DBBM, presenting with persistent intraoral swelling and an extraoral draining fistula that failed to resolve after repeated antibiotic therapy and was initially treated as a chronic infection [166]. These observations demonstrate possible adverse responses in selected cases but cannot establish their incidence or be generalized to well-integrated residual particles in otherwise successful grafted sites. They therefore do not indicate that residual material is generally harmful; rather, they show that biomaterial-specific and patient- or site-related factors can occasionally convert a structural remnant into a clinically relevant foreign body. Residual biomaterial should consequently be interpreted as a potential modulator of the local immune and remodeling microenvironment, not as an inherently pathological component.

From an implantological perspective, these immune-mediated and remodeling-related issues are particularly relevant at the bone–implant interface. If a substantial portion of the surrounding tissue contains components with limited biological activity, the adaptive capacity of this tissue under sustained functional loading may be reduced. It has therefore been hypothesized that such conditions could influence long-term implant stability, although this relationship remains difficult to verify clinically because implant outcomes depend on multiple biological, mechanical, surgical, and patient-related factors [29,131,167]. Similarly, some reports have suggested a potential association between residual graft material and increased susceptibility to complications such as peri-implantitis. However, current evidence does not establish a clear causal relationship between the presence of biomaterials and the occurrence of these complications [168,169,170]. These hypotheses should be distinguished from clinical evidence, which does not show that well-integrated residual particles generally compromise long-term implant outcomes.

It is therefore important to interpret residual graft persistence in a balanced manner. Materials with high structural stability may effectively preserve the volume of the augmented site and support favorable clinical outcomes [68]. Several clinical studies report long-term stability of regenerated bone and implants, even in the presence of residual biomaterial [29,168,171]. Thus, residual graft persistence should not be interpreted as treatment failure or reduced biological quality in itself. Different biomaterials may achieve distinct biological endpoints while still supporting durable implant treatment. The clinical significance of residual material likely depends on the specific biomaterial, intended therapeutic objective, quantity and maturity of newly formed bone, graft integration with host bone, amount and distribution of residual particles, and long-term clinical performance [68,168].

For indications in which progressive graft replacement is a central objective, these observations highlight the need for biomaterials that support physiological remodeling while maintaining adequate structural stability [88,96]. Modern regenerative strategies are therefore no longer focused solely on achieving sufficient bone volume, but also on promoting biological integration, active remodeling, and long-term functional adaptation [68,96]. Accordingly, increasing attention is being directed toward materials capable of interacting with the host environment and supporting not only bone formation, but also its subsequent remodeling and maintenance [88,96]. This objective should not be generalized to indications in which controlled scaffold persistence is clinically advantageous.

## 6. Assessment Methods for Bone Remodeling and Regenerated Bone Quality

The assessment of bone augmentation outcomes should not be limited to determining whether the defect has been filled or whether the augmented volume has been preserved. In the context of the biological quality of regenerated bone, it is essential to determine the proportion of newly formed vital bone, residual graft material, connective tissue, marrow spaces, and other non-mineralized components within the regenerated area [118,119]. For this reason, evaluation of bone regeneration commonly relies on histological and histomorphometric analyses, together with radiological and three-dimensional imaging techniques, while biological and immunohistochemical methods may provide additional information in selected experimental or translational settings [105,172]. Each of these approaches provides different information and has specific limitations; therefore, their results should be interpreted in a complementary manner [105].

### 6.1. Histological and Histomorphometric Assessment

Histological and histomorphometric analyses are among the most important methods for evaluating the quality of tissue formed after bone augmentation. They allow for the direct assessment of the composition of the regenerated area, including the percentage of newly formed bone, the amount of residual graft material, and the proportion of connective tissue or marrow spaces [118,119]. These parameters make it possible to determine whether the preserved or regenerated volume primarily reflects new bone formation or the persistence of non-resorbed biomaterial particles [118].

Histomorphometric evaluation may also include the assessment of bone-to-biomaterial contact and the degree of integration between the graft material and newly formed tissue [117,153]. These parameters are particularly relevant for osteoconductive materials, whose regenerative function depends largely on the provision of a surface for cell migration and bone deposition. However, bone-to-biomaterial contact should not be interpreted as evidence of complete biological remodeling. A biomaterial may be structurally integrated with newly formed bone while remaining only partially resorbed and showing limited participation in physiological bone turnover [29,118].

The main limitations of histological assessment include its invasive nature and the fact that analysis is usually based on a small biopsy obtained from a specific region and at a specific time point. Therefore, the findings may not fully reflect the entire augmented area or the dynamic nature of remodeling over time [153]. Despite these limitations, histology and histomorphometry remain key methods for assessing the biological quality of regenerated bone [118,119].

### 6.2. Radiological and Three-Dimensional Imaging Assessment

Radiological imaging techniques, including conventional radiography, CT, cone-beam CT (CBCT), and micro-CT, are widely used to evaluate bone augmentation outcomes [105,173]. These methods enable assessment of volumetric changes, contour stability, radiographic density, and the spatial organization of the augmented area [174,175]. In clinical practice, CBCT is particularly useful for evaluating alveolar ridge dimensions, graft height and width, and the amount of tissue available for implant planning [173].

Radiological methods are especially valuable for monitoring volumetric stability over time. They allow for the evaluation of dimensional changes, contour collapse, graft volume maintenance, and changes in radiographic density [176,177]. However, one of their major limitations is the inability to reliably distinguish newly formed bone from residual graft material, particularly when the biomaterial is highly radiopaque [105,178]. In such cases, radiological findings may suggest a favorable regenerative outcome, whereas histological analysis may reveal that a substantial proportion of the augmented volume still consists of residual graft particles [118,179].

Therefore, radiological stability should not be automatically equated with complete biological regeneration. Imaging techniques provide important information about volume and structure, but they do not directly assess tissue vitality, cellular activity, vascularization, or the actual participation of regenerated bone in physiological remodeling [105,180].

### 6.3. Biological and Immunohistochemical Assessment

Biological assessment methods, including immunohistochemical analyses and the evaluation of selected molecular or biochemical markers, may complement conventional histological and radiological approaches. These methods can provide more detailed information about biological processes occurring within regenerated tissue, such as osteoblastic and osteoclastic activity, angiogenesis, matrix mineralization, and local inflammatory or immune responses [181,182]. In this context, markers related to osteogenesis, bone remodeling, vascularization, and macrophage activity may be useful for assessing the biological activity of the regenerated area, particularly in experimental, preclinical, or translational studies [183,184].

However, such analyses are not routinely used in clinical studies in humans. Their application requires appropriate biopsy material, additional laboratory procedures, and standardized result interpretation. Therefore, in the clinical evaluation of bone grafting materials, biological and immunohistochemical methods should be regarded as complementary tools rather than primary outcome measures [172,181]. Their value lies in providing information on tissue activity and remodeling potential that cannot be obtained from radiological imaging or conventional histomorphometry alone [181,182].

### 6.4. Limitations in Outcome Interpretation

The interpretation of bone regeneration outcomes is complicated by the heterogeneity of available studies. Differences in graft material, surgical procedure, defect location, healing time, biopsy site, imaging protocol, and outcome reporting make direct comparisons between studies challenging [119,185]. Commonly reported parameters include the percentage of newly formed bone, residual graft material, connective tissue or marrow spaces, volumetric changes, and radiographic density; however, these outcomes are not always measured or reported in a standardized manner [119,185].

Another important limitation is the lack of a universally accepted definition of vital bone and the absence of standardized criteria for assessing the functional quality of regenerated tissue [117,120]. A high percentage of newly formed bone does not necessarily indicate full maturation or long-term remodeling capacity, just as the presence of residual biomaterial does not automatically indicate treatment failure [118,186]. The clinical relevance of these findings depends on multiple factors, including material properties, healing time, host biology, defect characteristics, and local biomechanical conditions [119,187].

For this reason, the evaluation of biomaterials should integrate both quantitative and qualitative outcomes. Particular attention should be given to the relationship between newly formed bone, residual graft material, volumetric stability, and remodeling potential [118,119]. Such an approach provides the basis for comparing different biomaterials in terms of their ability to support the formation offunctional, biologically active bone tissue capable of remodeling.

No single assessment modality captures all five domains of bone remodeling dynamics. Histology and histomorphometry characterize tissue composition and graft integration with host bone, imaging documents three-dimensional and temporal changes in volume, and biological or immunohistochemical analyses can provide information on vascular, osteogenic, osteoclastic, and immune activity. Demonstrating subsequent physiological turnover and functional adaptation additionally requires longitudinal assessment after implant placement and loading. Accordingly, multimodal evaluation across standardized time points is needed to distinguish early tissue formation from durable functional regeneration [105,119,172,181,182].

## 7. Comparative Evidence on Bone Remodeling After Augmentation with Different Biomaterials

Following the discussion of assessment methods, this section summarizes clinical, radiological, and histological evidence on bone remodeling after augmentation with different biomaterials. The available studies indicate that grafting materials differ not only in clinical outcomes, but also in the extent of newly formed bone, residual graft persistence, the proportion of connective tissue or marrow spaces, dimensional stability, and remodeling dynamics.

Therefore, the following subsections compare the main groups of biomaterials with regard to their ability to support new bone formation, undergo replacement by host bone, and maintain the augmented volume. This comparison provides the basis for evaluating whether specific materials primarily support persistence-oriented space maintenance or a replacement-oriented remodeling pattern characterized by progressive replacement by newly formed bone. Because biological adjuncts modify the behavior of a carrier rather than constitute an independent graft category, functionalized constructs are compared separately in Section 7.4.

Consistent with the five-domain framework defined in Section 2, the following comparisons consider the temporal progression of new vital bone formation, vascular ingrowth, graft integration with host bone, graft resorption, remodeling, or persistence, and subsequent physiological turnover and functional adaptation whenever these outcomes were reported. Because human studies most frequently report histomorphometric and dimensional endpoints, uneven coverage of these domains reflects limitations of the available evidence rather than a change in the analytical framework.

### 7.1. Autogenous Bone Grafts Versus Xenogeneic Bone Substitutes

Autogenous bone grafts and xenogeneic bone substitutes represent two distinct regenerative strategies. Autogenous bone provides a biologically active grafting material, whereas xenogeneic substitutes primarily act as osteoconductive scaffolds with high structural stability. Therefore, their comparison is useful for distinguishing between replacement-oriented remodeling and persistence-oriented maintenance of the augmented volume.

In a network meta-analysis (NMA) by Al-Moraissi et al., including 52 randomized controlled trials (RCTs) and 1483 biopsies after maxillary sinus augmentation, autogenous bone showed favorable histomorphometric outcomes, particularly with regard to new bone formation and residual graft content. At healing times shorter than 6 months, new bone formation was higher for autogenous bone than for xenografts, with a weighted mean difference of 7.93 percentage points. At healing times of 6 months or longer, residual graft content was lower for autogenous bone than for xenografts, with a weighted mean difference of 14.62 percentage points [123]. These findings indicate that autogenous bone remains among the most biologically active grafting materials, particularly with regard to early new bone formation and limited residual graft persistence.

However, the performance of a biomaterial should not be interpreted independently of local clinical conditions. In a subsequent NMA including 67 studies, 1955 patients, and 2405 sinus augmentation procedures, Khijmatgar et al. showed that the ranking of materials for new bone formation depended on residual bone height. When residual bone height was below 4 mm, the highest-ranked materials were combinations of xenografts with allografts, xenografts with alloplasts, and autogenous bone. In contrast, when residual bone height was 4 mm or greater, autogenous bone ranked highest [188]. This suggests that comparisons between autogenous bone and xenogeneic substitutes should also take anatomical conditions into account.

Direct clinical comparisons generally support the biological advantage of autogenous bone in terms of new bone formation. In an RCT by Schmitt et al., after 5 months of healing, the bone volume fraction of newly formed bone was highest in the autogenous bone group, reaching 41.74 ± 2.10%, compared with 24.90 ± 5.67% for anorganic bovine bone. In the xenogeneic group, residual bone substitute material accounted for 21.36 ± 4.83% [189]. These data support the role of autogenous bone as a biological reference material, while also showing that xenogeneic substitutes may remain within the regenerated tissue as residual scaffold particles.

An important interpretative limitation is that similar amounts of hard tissue do not necessarily indicate the same biological composition. Correia et al. compared autogenous bone with a porcine xenograft after 6 months of healing and reported comparable proportions of total hard tissue in both groups: 57.31 ± 2.91% for autogenous bone and 56.01 ± 2.86% for the xenograft. However, the histological composition differed between groups. Autogenous bone was described as almost completely resorbed and integrated with newly formed vital bone, whereas residual xenogeneic particles were still observed in contact with vital bone [190]. This indicates that total hard tissue should not be automatically equated with newly formed bone.

A similar issue applies to composite grafts containing both autogenous and xenogeneic components. De Vicente et al. evaluated sinus augmentation using autogenous bone combined with bovine-derived HA and reported a mean of 29% newly formed bone and 21% residual anorganic bovine bone [191]. Thus, even in composite grafts, the xenogeneic component may persist as a residual scaffold.

The addition of autogenous bone to a xenogeneic scaffold does not always result in a significant increase in new bone formation. Pikdöken et al. compared bovine bone mineral alone with a mixture of autogenous cortical bone scrapings and bovine bone mineral in a 1:4 ratio. After 4 months of healing, newly formed bone accounted for 25.73% in the mixed-graft group and 24.19% in the bovine bone mineral group, with no statistically significant difference [192]. This suggests that the effect of adding autogenous bone may depend on the proportion of the autogenous component, healing time, and the local regenerative environment.

The relevance of the ratio between autogenous and xenogeneic components was further illustrated by Mordenfeld et al., who compared mixtures of DBBM and autogenous bone in ratios of 60:40 and 90:10. After 7.5 months, the 60:40 mixture resulted in greater ridge width gain than the 90:10 mixture, reaching 3.5 ± 1.3 mm and 2.9 ± 1.3 mm, respectively, and showed less graft width reduction (37.0 ± 19.9% versus 46.9 ± 23.5%). However, no significant histomorphometric differences were found between the groups [193]. These findings suggest that a higher proportion of autogenous bone may improve dimensional stability, but does not necessarily translate into significantly greater new bone formation.

Pereira et al. also showed that adding autogenous bone to DBBM (Bio-Oss^®^) did not consistently increase new bone formation in all regions of the augmented sinus. After 6 months of healing, autogenous bone showed new bone formation values of 37.8%, 38.1%, and 44.5% in the pristine bone, intermediate, and apical regions, respectively. The corresponding values for DBBM were 33.4%, 32.5%, and 34.3%, whereas DBBM combined with autogenous bone showed 32.8%, 36.1%, and 27.8% [129]. These findings indicate that the biological response depends not only on the addition of an autogenous component, but also on the biomaterial type and the region analyzed.

From a clinical perspective, xenogeneic components may contribute to volume stability. In a systematic review and meta-analysis, Starch-Jensen et al. reported that volumetric reduction occurred regardless of the material used, but that autogenous bone showed improved volumetric stability when mixed with a non-resorbable xenograft compared with autogenous bone alone [42]. This indicates that residual xenogeneic particles may be clinically useful for volume maintenance while representing a persistence-oriented remodeling pattern.

Taken together, the available evidence indicates that autogenous bone and xenogeneic substitutes should not be arranged into a simple hierarchy of universal superiority. Autogenous bone generally shows greater biological activity, particularly during early healing, and is associated with a lower proportion of residual graft material. Xenogeneic substitutes, in contrast, may support volume maintenance, but their persistence can influence outcomes reported as total hard tissue. Therefore, in the evaluation of bone regeneration, it is essential to distinguish between total hard tissue, newly formed vital bone, and residual xenogeneic material.

### 7.2. Allogeneic Grafts Versus Xenogeneic Bone Substitutes

Direct comparisons between allogeneic and xenogeneic grafts remain heterogeneous because both categories encompass materials with different processing methods, physical forms, resorption profiles, and clinical indications. Available evidence indicates that both can support implant-site development, but their relative performance differs across the remodeling domains considered in this review, including vital bone formation, residual graft persistence, integration, dimensional stability, and subsequent turnover.

In the systematic review by Abushama et al., xenografts and allografts used before dental implant placement were compared across studies published between 2016 and 2024. The review included adult patients undergoing oral and maxillofacial surgical procedures prior to implant rehabilitation. Although the included evidence was heterogeneous, the authors indicated that both allogeneic and xenogeneic materials may be clinically effective, while clear superiority of one category over the other remains difficult to demonstrate because of differences in indications, study designs, graft processing, and outcome measures [194].

Histomorphometric data from maxillary sinus augmentation suggest that allogeneic grafts may, under some conditions, support greater new bone formation than xenogeneic bovine mineral. In the RCT by Schmitt et al., after 5 months of healing, newly formed bone accounted for 35.41 ± 2.78% in the mineralized cancellous bone allograft group, compared with 24.90 ± 5.67%in the anorganic bovine bone group [189]. These findings suggest that a human-derived mineralized matrix may support more active bone formation than a slowly resorbing xenogeneic scaffold under sinus augmentation conditions. At the same time, the allograft did not reach the values observed for autogenous bone, indicating that donor-derived bone matrices should not be considered biologically equivalent to autogenous grafts.

Additional data from maxillary sinus augmentation indicate that allogeneic and xenogeneic materials may also differ in the expression of remodeling markers. Kungvarnchaikul et al. compared deproteinized bovine bone (DBB) with FDBA in two-stage maxillary sinus floor augmentation. Six months after augmentation, the FDBA group showed higher expression of the genes encoding runt-related transcription factor 2 (RUNX2), osteopontin (OPN), and receptor activator of nuclear factor-κB ligand (RANKL), as well as greater OPN immunoreactivity, than the DBB group [195]. These findings suggest a more active biological response with the allograft, although they are based primarily on molecular and immunohistochemical parameters rather than conventional histomorphometric outcomes.

In alveolar ridge preservation, the relationship between allogeneic and xenogeneic materials appears less consistent. Stumbras et al. compared natural bovine bone mineral covered with a collagen membrane with FDBA covered with a collagen membrane in extraction sockets in the anterior maxilla. After 3 months, the bovine bone mineral group showed more favorable dimensional preservation than the allograft group, particularly with regard to horizontal ridge width reduction [196]. This may reflect the greater structural persistence of xenogeneic particles, which can favor early dimensional preservation.

This observation is supported by broader evidence from socket grafting studies. In the systematic review by Jambhekar et al., including RCTs on flapless extraction and socket grafting, xenografts were associated with the lowest mean buccolingual ridge width loss at the crest, reaching 1.3 mm. For allografts, this value was 1.63 mm. Mean buccal wall height loss was very similar between the two groups, reaching 0.57 mm for xenografts and 0.58 mm for allografts. Histological outcomes also did not clearly favor allografts, as mean vital bone content was 35.72% for xenografts and 29.93% for allografts, whereas residual graft material accounted for 19.3% and 21.75%, respectively [197].

Similar conclusions can be drawn from the systematic review by De Risi et al., which assessed histological and histomorphometric outcomes after alveolar ridge preservation. The highest percentage of newly formed bone after 3 months was observed in procedures using allografts and reached 54.4%, whereas the lowest value was reported after 5 months in procedures using xenografts, reaching 23.6%. However, the authors did not identify statistically significant differences among the evaluated procedures [198]. These data suggest that allografts may show favorable new bone values in some settings, but this finding should not be interpreted as consistent superiority over xenogeneic materials.

Recent randomized human histological evidence further demonstrates the importance of distinguishing among allograft preparations rather than treating DFDBA as a single uniform material. Foster et al. enrolled 120 patients requiring extraction of a non-molar tooth and randomized them to particulate DFDBA alone, DFDBA fibers alone, or either form combined with xenograft and obtained histological samples after 18–20 weeks. Vital bone accounted for 37.33% with particulate DFDBA and 40.76% with DFDBA fibers, compared with 24.46% and 23.85% in the respective xenograft-containing combinations. DFDBA fibers also left substantially less residual allograft than particulate DFDBA, both when used alone (3.57% versus 16.25%) and when combined with xenograft (2.19% versus 9.88%), while dimensional ridge changes did not differ significantly among groups [56]. These findings show that similar dimensional outcomes may coexist with markedly different histological compositions and that both processing and physical form influence graft turnover.

Additional contemporary human histological evidence further supports the product- and context-specific interpretation of allogeneic graft behavior. In a randomized clinical trial by Zampara et al., extraction sockets grafted with freeze-dried cancellous allograft showed 56.0 ± 12.8% vital bone and 4.5 ± 7.7% residual graft after 3 months, compared with 17.2 ± 12.8% vital bone and 22.2 ± 7.7% residual graft in the xenograft group [199]. In a cohort study by Iezzi et al., FDBA-treated sockets showed 35.22 ± 10.79% newly formed bone, 12.41 ± 7.87% residual graft, and 64.61 ± 27.14% bone-to-biomaterial contact after 3 months [200]. In the related follow-up case series by Barone et al., new bone bridges were observed around residual FDBA particles without inflammatory infiltrates, while all implants placed without additional augmentationremained successful at 1 and 2 years [201]. Together, these findings indicate that allogeneic grafts may support substantial vital bone formation and integration, while residual particles can coexist with successful long-term clinical performance.

The NMA by Canullo et al., including 88 RCTs, 2805 patients, and 3073 sockets, further highlights the discrepancy between dimensional preservation and histological outcome. In this analysis, xenografts and allografts were among the most predictable materials for preserving horizontal and vertical ridge dimensions. At the same time, xenografts, despite favorable dimensional outcomes, were associated with a histological profile characterized by greater graft persistence [202]. This indicates that dimensional preservation and histological composition are distinct outcome domains and should not be ranked interchangeably.

The variability of histological outcomes was also emphasized by Chan et al., who analyzed bone quality after socket preservation using grafting materials. The authors reported that the effect of grafting on vital bone formation was heterogeneous, and that, in the case of xenogeneic materials, residual particles may persist for several months and substantially influence the histological composition of the healed site. Therefore, outcomes based only on ridge dimensions or total hard tissue may overestimate the extent of biological regeneration [203].

Evidence from the systematic review by Avila-Ortiz et al. confirms the clinical relevance of both material groups in alveolar ridge preservation. The authors showed that socket filling with grafting material limits post-extraction ridge volume loss compared with extraction alone. Subgroup analyses indicated that both xenografts and allografts were associated with favorable dimensional outcomes, particularly in the preservation of mid-buccal and mid-lingual ridge height [204].

In the analysis by Al-Moraissi et al., which included 52 RCTs and 1483 biopsies, the combination of allograft and xenograft ranked highest for newly formed bone, whereas xenogeneic materials showed greater residual graft persistence, particularly at longer healing times [123].

Similarly, Khijmatgar et al., in a NMA including 67 studies, 1955 patients, and 2405 sinus augmentation procedures, showed that the ranking of biomaterials depended on residual bone height. When residual bone height was below 4 mm, xenograft combined with allograft ranked among the most favorable materials for new bone formation. In contrast, when residual bone height was 4 mm or greater, autogenous bone and combinations involving xenografts ranked highest [188]. These findings indicate that allogeneic and xenogeneic materials should not be compared as fixed, isolated categories, but in relation to anatomical conditions, healing time, and whether they are used alone or in combination.

Taken together, allogeneic and xenogeneic materials should not be placed in a fixed hierarchy of biological superiority. Selected modern allografts, including aseptically processed DFDBA, may support substantial vital bone formation and comparatively active graft replacement, whereas slowly resorbing xenografts may offer superior or more predictable dimensional maintenance. These outcomes describe different regenerative domains rather than mutually exclusive definitions of success. The most appropriate material therefore depends on the clinical indication and desired balance among vital bone formation, vascular and structural integration, controlled graft persistence or resorption, volume maintenance, and long-term physiological remodeling.

### 7.3. Synthetic and Alloplastic Biomaterials Versus Xenogeneic Bone Substitutes

Synthetic and alloplastic biomaterials, particularly calcium phosphate-based materials such as HA, β-TCP, BCP, nanohydroxyapatite (nHA), and modified HA formulations, have been evaluated as alternatives to xenogeneic bone substitutes. Their comparison is especially relevant because selected synthetic materials may show comparable or higher new bone formation and lower residual graft persistence, representing a more replacement-oriented histological profile when progressive graft replacement is the intended therapeutic endpoint.

In maxillary sinus augmentation, several studies indicate that BCP may achieve histomorphometric outcomes comparable to or slightly more favorable than anorganic bovine bone. In the RCT by Schmitt et al., after 5 months of healing, newly formed bone accounted for 30.28 ± 2.16% in the BCP group and 24.90 ± 5.67% in the anorganic bovine bone group. Residual graft material was also lower for biphasic calcium phosphate, reaching 15.80 ± 2.10%, compared with 21.36 ± 4.83% for anorganic bovine bone [189]. These data suggest that BCP may undergo more extensive replacement by newly formed bone than slowly resorbing bovine-derived xenograft particles under sinus augmentation conditions.

Comparable findings were reported in an RCT by Tomas et al., which evaluated injectable BCP and anorganic bovine bone in human alveolar bone defects. After 6 months, newly formed bone reached 39.91 ± 8.49% in the BCP group and 41.73 ± 13.99% in the anorganic bovine bone group. Residual biomaterial accounted for 28.61 ± 11.38% and 31.72 ± 15.52%, respectively, while soft tissue represented 31.49 ± 11.09% in the BCP group and 26.54 ± 7.25% in the anorganic bovine bone group. No statistically significant differences were found for newly formed bone, residual biomaterial, or soft tissue [205]. These findings indicate that injectable BCP may achieve a histological outcome similar to anorganic bovine bone, with a slightly lower proportion of residual material.

However, calcium phosphate-based materials do not always outperform xenogeneic substitutes. Kurkcu et al. compared anorganic bovine-derived HA with β-TCP in sinus augmentation. After a mean healing time of 6.5 months, newly formed bone was significantly higher in the bovine-derived HA group, reaching 30.13 ± 3.45%, compared with 21.09 ± 2.86% in the β-TCP group. Residual graft particle area was 31.88 ± 6.05% for bovine-derived HA and 34.05 ± 3.01% for β-TCP, while soft tissue accounted for 37.99 ± 5.92% and 44.86 ± 4.28%, respectively [61]. These findings show that β-TCP, despite being synthetic and resorbable, may not necessarily produce greater early bone formation than xenogeneic HA.

This variability is also visible in studies evaluating synthetic HA-based materials. In the split-mouth RCT by Stacchi et al., sintered nHA was compared with anorganic bovine xenograft in maxillary sinus grafting. Newly formed bone was reported at 34.9% for nHA and 38.5% for the bovine xenograft, whereas residual graft material accounted for 20.6% and 22.3%, respectively [206]. These values indicate broadly comparable remodeling patterns, with a slightly higher new bone percentage for the xenograft and a slightly lower residual material percentage for nHA.

Other studies have shown that synthetic calcium phosphate substitutes may still persist in considerable amounts, depending on formulation and healing time. Tosta et al. evaluated a synthetic bone substitute for maxillary sinus grafting and reported 33.70% newly formed bone, 32.88% residual graft material, and 33.42% soft tissue [207]. This indicates that synthetic materials should not be automatically interpreted as rapidly replaced by host bone; in some clinical settings, they may remain as a substantial residual scaffold, similarly to xenogeneic substitutes.

In the systematic review and NMA by Somngamet al., 11 RCTs including 283 patients and 362 sinus augmentations were analyzed to compare BCP with other bone substitute materials. Autogenous bone produced 12.33 percentage points more new bone formation than BCP, while allografts showed5.14 percentage points more new bone formation than BCP. In contrast, xenografts showed 4.14 percentage points less new bone formation than BCP. In the same analysis, residual graft material was highest in xenografts, with a difference of 6.21 percentage points [208]. These findings indicate that, among non-autogenous materials evaluated for sinus augmentation, BCP showed a more replacement-oriented histological profile than xenografts, with higher new bone formation and lower residual graft persistence in the network comparison. The clinical relevance of this profile remains indication-dependent.

At the same time, volumetric outcomes may favor xenogeneic materials. In the systematic review by Pesce et al., which included 21 RCTs, xenografts showed the lowest volume reduction after 6 months of healing, with graft shrinkage of 7.30 ± 15.49%. In contrast, autogenous grafts showed the highest reduction, reaching 41.71 ± 12.63% [209]. Although this comparison was not limited to calcium phosphate biomaterials, it supports an important interpretation: slow remodeling and persistence of xenogeneic particles represent a distinct histological endpoint that may be advantageous for volume maintenance in sinus augmentation.

Data from extraction socket and ridge preservation studies provide additional evidence that synthetic materials may show lower residual persistence than xenografts, often with more favorable vital bone formation. Molly et al. evaluated biomaterials placed in extraction sockets and reported that bovine porous bone mineral showed the greatest percentage of residual biomaterial after 6 months, reaching 20.2 ± 17.0%. In comparison, the synthetic sponge based on polylactic–polyglycolic acid technology showed only 5.6 ± 8.9% residual material, while the coral-derived calcium carbonate material showed 12.0 ± 16.4% [210]. These results suggest that selected synthetic or alloplastic substitutes may undergo faster degradation or replacement than xenogeneic bovine mineral in socket healing.

This pattern was further supported by Casarez-Quintana et al., who compared a composite bovine-derived xenograft with a synthetic HA-sugar cross-linked collagen matrix for ridge preservation. After 16 weeks of healing, the HA alloplast group showed significantly more vital bone formation than the bovine-derived xenograft group, reaching 39.3 ± 17.8% and 26.8 ± 15.8%, respectively. The xenograft group showed approximately 18% residual graft particles, while the alloplast group showed no residual graft particles [211]. These findings support the interpretation that selected alloplastic matrices may exhibit a more replacement-oriented histological profile than bovine-derived xenogeneic scaffolds under these conditions.

The dimensional data from the same study confirm that higher vital bone formation does not necessarily translate into better ridge preservation. The change in buccal ridge height was −0.7 ± 1.7 mm in the bovine xenograft group and −0.2 ± 1.6 mm in the HA alloplast group. The change in lingual ridge height was −0.9 ± 1.3 mm and −1.1 ± 1.6 mm, respectively. The change in ridge width 4 mm apical to the crest was −0.9 ± 1.7 mm for the bovine xenograft and −1.8 ± 1.9 mm for the synthetic HA matrix, with no statistically significant difference [211]. These findings indicate that higher vital bone formation must be interpreted separately from dimensional stability.

The biological behavior of calcium phosphate materials may also depend strongly on healing time. Canullo et al. evaluated magnesium-enriched HA in extraction sockets at different healing intervals. Histomorphometric analysis showed 15.1 ± 16.7% regenerated bone after 2 months and 77.4 ± 14.9% after 4 months. During the same period, residual graft material decreased from 21.7 ± 15.4% to 11.6 ± 15.7%, while connective tissue or marrow space decreased from 63.3 ± 22.4% to 11.0 ± 6.9% [212]. These data show that some synthetic HA-based materials may undergo substantial remodeling within a relatively short healing interval, although the magnitude of change may depend on the material formulation and defect environment.

Other alloplastic materials may also show favorable remodeling characteristics. Bioactive glass, for example, has been evaluated as a synthetic grafting material because of its osteoconductive and surface-reactive properties. In socket preservation, Froum et al. reported that sites grafted with bioactive glass showed 59.5% vital bone and 5.5% residual graft material after 6 to 8 months of healing. In comparison, sites treated with DFDBA showed 34.7% vital bone and 13.5% residual graft material, while naturally healed control sites showed 32.4% vital bone [213]. These findings illustrate that selected alloplastic materials may support substantial vital bone formation with relatively limited residual graft persistence.

Recent data from GBR also suggest that newer bioceramic materials may achieve dimensional performance comparable to xenografts. In the systematic review by Bozza et al., a multicenter randomized trial comparing a novel bioceramic with a bovine-derived xenograft was summarized. Immediately postoperatively, horizontal bone thickness was 6.053 ± 1.262 mm in the bioceramic group and 5.907 ± 1.269 mm in the bovine xenograft group. After 180 ± 14 days, the values were 5.775 ± 1.345 mm and 5.273 ± 1.285 mm, respectively [185]. These results suggest that selected synthetic bioceramics may provide stable dimensional outcomes comparable to xenogeneic substitutes, although the evidence base remains smaller than for conventional xenografts.

Taken together, the available evidence indicates that synthetic and alloplastic biomaterials, especially BCP and selected HA-based matrices, may show a more replacement-oriented histological profile than xenogeneic substitutes, characterized by similar or higher new bone formation and lower residual biomaterial persistence. This pattern is material- and indication-dependent and should not be generalized to all synthetic substitutes, since β-TCP and some other formulations may show lower early bone formation or substantial residual scaffold persistence. A replacement-oriented histological profile should not be interpreted as universal biological or clinical superiority over a stable, well-integrated scaffold. Therefore, regenerative outcomes should distinguish among new bone formation, graft replacement or persistence, dimensional maintenance, and the intended therapeutic objective.

### 7.4. Biologically Functionalized Grafts Versus Unmodified Scaffolds

Biological functionalization differs conceptually from comparisons based only on graft origin because the intervention consists of both a carrier and an incorporated signaling molecule, matrix modifier, or cellular component. A valid comparison should therefore treat the functionalized construct as a distinct intervention and, whenever possible, use an otherwise identical unmodified carrier as the control. This distinction is essential for determining whether an adjunct changes the amount and maturation of vital bone, vascular ingrowth, graft integration with host bone, graft resorption or persistence, and subsequent physiological turnover, rather than merely reflecting the intrinsic behavior of the scaffold.

The broadest clinical synthesis is provided by the American Academy of Periodontology best-evidence systematic review, which included 39 articles on autologous blood-derived products, enamel matrix derivative, rhPDGF-BB, and rhBMP-2 in alveolar ridge preservation or reconstruction and implant-site development [37,38]. Most ridge-preservation and reconstruction studies reported clinical or radiographic outcomes similar to conventional protocols, although selected biologics improved early histomorphometric findings or wound healing. In alveolar ridge and maxillary sinus augmentation, superiority in clinical or radiographic outcomes was not consistently demonstrated, even when earlier bone formation was observed. These findings indicate that biological functionalization may modify the kinetics or early quality of healing without necessarily changing the final dimensional result or proving a durable remodeling advantage.

For rhBMP-2, the most direct evidence of an early histological effect comes from the randomized clinical study by Sun et al. [105]. Forty patients undergoing extraction of a single-rooted tooth were allocated to low-dose rhBMP-2 incorporated into a biomimetic calcium-phosphate coating on β-TCP, β-TCP alone, or spontaneous healing. At 6 weeks, newly formed bone accounted for 21.18 ± 7.62%, 13.44 ± 6.03%, and 9.49 ± 0.08%, respectively, while residual material represented 10.04 ± 4.57% in the rhBMP-2 construct and 20.60 ± 9.54% with β-TCP alone. The comparison supports the capacity of controlled rhBMP-2 delivery to increase the amount of newly formed bone during early healing and accelerate carrier replacement. However, a 6-week biopsy cannot determine whether the additional woven bone matures into organized lamellar bone, remains stable after implant loading, or undergoes physiological turnover comparable to native tissue.

More recent pilot evidence reinforces the distinction between early dimensional benefit and long-term biological success. Thammanichanon et al. randomized 11 participants with 16 extraction sites to an absorbable collagen sponge loaded with rhBMP-2 or spontaneous healing [214]. At 12 weeks, the functionalized group showed 1.80 mm less reduction in buccal bone height, but no significant difference in palatal or lingual height reduction or overall buccolingual ridge-width change. This finding is clinically relevant, yet the small sample, short observation period, and absence of histological or post-loading outcomes prevent conclusions about bone vitality, graft replacement, and long-term remodeling.

Taken together, rhBMP-2 may contribute to bone vitality by recruiting and directing osteoprogenitor cells and may shorten the interval to early bone formation. Its effect nevertheless remains inseparable from dose, carrier affinity, release kinetics, and spatial retention. A signal released too rapidly may be lost from the defect, whereas excessive or poorly confined exposure may produce an exaggerated local response or bone formation outside the intended compartment [34,35,36]. Consequently, an early increase in bone fill cannot be generalized to all rhBMP-2 constructs, and long-term benefit should be established using matched-carrier controls, adverse-event reporting, and follow-up after functional loading [37,38,214].

BMP-7 represents a related but substantially less mature strategy. Its osteogenic signaling suggests potential to promote progenitor-cell differentiation and bone matrix formation, but the available evidence for localized alveolar augmentation remains predominantly preclinical or derived from early clinical applications [106,107]. The absence of robust human comparisons with identical unmodified scaffolds and of long-term histological or implant-related follow-up means that a contribution to durable vital bone and physiological remodeling remains hypothetical. BMP-7 should therefore not be treated as clinically interchangeable with rhBMP-2 or described as an established enhancer of long-term augmentation success.

rhPDGF-BB acts through a different biological pathway. Rather than directly inducing osteogenic differentiation, it promotes chemotaxis and proliferation of reparative cell populations and may facilitate vascularized tissue integration. Tavelli et al. reviewed 63 human clinical studies with a mean follow-up of 10.7 ± 3.3 months and reported favorable outcomes when rhPDGF-BB was combined with allografts or xenografts for guided bone regeneration and alveolar ridge preservation, without serious treatment-related adverse events [108]. Nevertheless, the studies encompassed different indications, carriers, comparators, and endpoints, and most did not quantify long-term graft replacement or remodeling after implant loading. rhPDGF-BB may therefore improve the cellular and vascular conditions required for early healing, but current evidence does not establish that it consistently produces a larger amount of mature vital bone or a more completely remodeled augmented site.

Hyaluronic acid illustrates why biological functionalization should not be assumed to improve every regenerative outcome. As a hydrated extracellular-matrix component, it may support clot stabilization, graft handling, cell migration, and local angiogenic or inflammatory regulation. However, the 2024 systematic review and meta-analysis by Lorenzi et al., based on three randomized trials, found no statistically significant increase in new bone formation and no significant reduction in residual graft particles when hyaluronic acid was added to graft materials [109]. Thus, improvements in the healing microenvironment or handling characteristics do not necessarily translate into more vital bone or more complete scaffold replacement. Future assessment should distinguish among formulations, cross-linking methods, carrier materials, and indications rather than treating hyaluronic acid as a uniform osteogenic intervention.

Cellular functionalization is similarly dependent on the preparation and the carrier. In the study by Sauerbier et al., bovine bone mineral combined with autogenous bone was compared with bovine bone mineral combined with bone marrow aspirate concentrate (BMAC). After 3 to 4 months, new bone formation reached 14.3 ± 1.8% in the autogenous-bone combination and 12.6 ± 1.7% in the BMAC combination, while residual bovine mineral accounted for 31.3 ± 2.7% of the augmented tissue in the BMAC group [110]. These findings do not demonstrate an early histomorphometric advantage for BMAC and show that a substantial proportion of the functionalized construct may remain as residual scaffold. Because BMAC contains a heterogeneous and patient-dependent mixture of progenitor and hematopoietic cells and soluble mediators, its effect cannot be generalized across aspiration and processing protocols.

A 2025 Phase 2 randomized clinical trial provides a complementary perspective on a more standardized cellular strategy. Sanz et al. compared expanded autologous iliac-crest-derived mesenchymal stromal cells seeded on a synthetic bioabsorbable calcium-phosphate scaffold with autogenous bone blocks for three-dimensional augmentation of severely atrophic ridges [215]. Among 48 randomized patients, the cell-based intervention produced a greater short-term bone volume gain, with a mean between-group difference of 480.01 mm^3^, and implants were successfully integrated in both groups. Interpretation nevertheless requires caution: seven of 36 patients allocated to the cell-therapy group could not be treated because the required cell yield was not achieved, the trial was underpowered, and differences in the initially augmented volume could not be excluded as an explanation for part of the observed gain. The study demonstrates translational potential, but it does not yet establish superior histological maturity, physiological turnover, or long-term peri-implant stability.

The available comparisons therefore do not support a single hierarchy of biological adjuncts. BMP-2 primarily targets osteogenic commitment, rhPDGF-BB primarily supports cellular recruitment and vascularized repair, hyaluronic acid modifies the extracellular and handling environment, and BMAC or expanded mesenchymal cells provide variable cellular and paracrine inputs. Each approach may influence a different phase of healing, and an apparent benefit in one domain may coexist with no measurable improvement in another. For example, accelerated early bone formation may occur without better dimensional preservation, while improved volume may occur without evidence of more complete graft replacement or mature trabecular organization.

Biological functionalization could contribute to future bone vitality by increasing the recruitment, survival, and osteogenic differentiation of host or delivered cells and by supporting vascular supply during the vulnerable early phase of regeneration. Where scaffold replacement is the intended endpoint, a contribution to remodeling would require these early effects to progress toward coordinated scaffold resorption, formation of vascularized lamellar bone, and restoration of coupled osteoblastic and osteoclastic turnover. In indications prioritizing long-term space or contour maintenance, successful regeneration may instead include controlled persistence of a well-integrated scaffold alongside vital host bone. Long-term success would additionally require stable osseointegration, adaptation to mechanical loading, maintenance of peri-implant bone, and absence of delayed adverse responses. These sequential outcomes cannot be inferred from a single early biopsy, CBCT-derived volume, or short-term implant survival result.

From a comparative perspective, biologically functionalized constructs should therefore be evaluated as indication-, dose-, formulation-, and carrier-specific interventions. Current evidence supports the possibility of faster early healing or improved short-term bone formation with selected protocols, but it does not demonstrate that any of the reviewed adjuncts uniformly produces more vital, completely remodeled, and functionally durable bone than an appropriate unmodified scaffold [37,38,106,107,108,109,110,214,215]. Direct matched-carrier comparisons, standardized reporting across the five remodeling domains, and follow-up after implant loading are required to determine whether biological functionalization changes the long-term quality of regenerated bone rather than only the speed of its early formation.

Taken together, the available comparative evidence indicates that grafting materials differ substantially in the extent of new bone formation, residual material persistence, remodeling dynamics, and dimensional stability. These outcomes are influenced not only by the origin and composition of the biomaterial, but also by the surgical procedure, healing time, anatomical conditions, and method of assessment. A consolidated summary of the studies discussed in this section is provided in Table 2.

Whereas Table 2 summarizes the study-level comparative evidence and evidence syntheses discussed in this section and identifies their designs and healing intervals, Table 3 provides a category-level comparison of biological properties, representative human histomorphometric findings, remodeling behavior, clinical indications, and the quality and limitations of the available human evidence.

## 8. Limitations and Future Research Directions

Despite the growing number of studies on biomaterials used for bone augmentation, the interpretation of available evidence remains limited by considerable methodological heterogeneity. The studies differ in terms of surgical procedure, defect location, augmentation type, biomaterial category, graft combinations, healing time, and outcome assessment methods. Data are derived from maxillary sinus augmentation, alveolar ridge preservation, GBR, and horizontal or vertical ridge augmentation, which makes direct comparison of outcomes difficult. In addition, materials belonging to the same general category, such as xenografts, allografts, or synthetic substitutes, may differ substantially in composition, porosity, processing method, resorption pattern, and surface properties [185]. Therefore, results obtained for one specific material should not be automatically generalized to an entire biomaterial group.

Another important limitation is the lack of standardization in the timing of histological and radiological assessment. In some studies, biopsies are collected after 3–4 months, whereas in others they are obtained after 6 months or after longer healing periods. This is clinically relevant because biomaterials differ in their degradation and remodeling dynamics. A material that shows a high proportion of residual particles at an early time point may undergo further replacement by host bone during longer healing [212]. Conversely, a material that maintains volume may show limited remodeling even after extended observation [209]. Therefore, comparing histomorphometric outcomes without considering the timing of biopsy collection may lead to oversimplified conclusions regarding the regenerative potential of a given biomaterial.

A further challenge concerns the interpretation of parameters such as newly formed bone, vital bone, total hard tissue, and residual graft material. In many studies, the outcome reported as hard tissue may include both newly formed bone and remaining biomaterial particles. Consequently, a high proportion of hard tissue does not necessarily indicate a high proportion of biologically active, remodeling bone. This is particularly important for slowly resorbing materials, in which residual particles may remain within the regenerated area for a prolonged period [202,203]. At the same time, the presence of vital bone alone does not fully describe the quality of regeneration, because the percentage of vital bone does not provide complete information about bone maturity, mineralization, trabecular organization, vascularization, or the ability of the regenerated tissue to withstand functional loading after implant placement [105,126,127].

For this reason, one of the key limitations of the current evidence is the lack of a clear and widely accepted definition of functionally relevant bone within the augmented site. In clinical practice, the goal of treatment is not only to fill a defect or maintain volume, but to obtain tissue capable of implant integration and further physiological remodeling. However, most studies focus on histomorphometric parameters assessed at a single time point, often at the stage of implant bed preparation. Much less is known about whether a given proportion of vital bone, residual graft material, and connective tissue translates into primary and secondary implant stability, quality of osseointegration, peri-implant bone remodeling, marginal bone loss, and long-term implant success [203,216].

The effect of residual biomaterial on subsequent implant outcomes remains insufficiently understood. The presence of residual particles is not necessarily unfavorable, as it may help maintain the volume of the augmented area [88,213]. A high residual-material proportion may coexist with a lower relative proportion of vital bone within a biopsy; however, this compositional relationship does not by itself demonstrate impaired osseointegration or inferior clinical performance [42,62]. Available studies rarely allow for a clear determination of what amount or distribution of residual material is clinically neutral, beneficial, or unfavorable. Its significance should therefore be interpreted in relation to the specific material, therapeutic objective, amount and maturity of vital bone, integration, and long-term implant performance. Evidence also remains limited regarding whether xenogeneic, allogeneic, or synthetic residual particles have different implications for long-term function [75,162,165,217].

An additional limitation is that foreign body reactions and immune-mediated complications associated with residual biomaterials are not systematically assessed or reported in most clinical studies. Although such events appear to be uncommon, available case reports and case series indicate that persistent graft particles may, under unfavorable conditions, be associated with chronic inflammation, fibrotic tissue formation, foreign body giant cell reactions, graft particle migration or displacement, and the need for surgical removal or debridement [163,164,165,166,217]. Therefore, the absence of reported complications in many studies should not necessarily be interpreted as evidence that residual biomaterials are biologically inert. Rather, it reflects the fact that most available studies are not designed to evaluate delayed immune-mediated responses, foreign body granulomas, or long-term tissue reactions around residual particles. Future clinical studies should therefore include more detailed reporting of adverse biological responses, including chronic inflammation, foreign body reactions, graft migration, fibrotic encapsulation, and local bone loss, particularly in long-term follow-up after implant placement. Because these observations are predominantly case-based, they cannot establish the frequency of such events or the clinical significance of residual particles in general.

A related limitation is the relatively small number of long-term studies combining histological assessment with later clinical outcomes. Many publications analyze new bone formation, residual graft content, or dimensional changes of the ridge, but do not provide extended follow-up after implant loading. For a more complete evaluation of biomaterials, histomorphometric data should be correlated with parameters such as implant survival, implant success, marginal bone loss, biological complications, and peri-implant tissue stability. Such an approach would make it possible to determine whether a higher proportion of newly formed bone results in better functional outcomes, or whether, in certain clinical situations, volume maintenance and structural stability of the augmented site may be sufficient [203,210]. Accordingly, complete graft replacement should be regarded as one possible endpoint when biological replacement is the intended therapeutic objective, rather than as a universal prerequisite for long-term clinical success.

Future studies should therefore aim for greater standardization of biomaterial assessment. Standardized biopsy time points, consistent histomorphometric definitions, and uniform reporting of newly formed bone, residual biomaterial, connective tissue, and marrow spaces would improve comparability between studies. It would also be valuable to combine histological methods with radiological imaging, volumetric analysis, bone quality assessment, and post-implant placement clinical outcomes. Such an approach would help distinguish materials that primarily maintain space from those that more effectively support biological remodeling and replacement by host bone. For allogeneic materials in particular, future trials should report the tissue-bank source, donor- and processing-related variables, degree of demineralization, aseptic processing versus terminal sterilization, graft architecture, and particle or fiber form, and should relate product-specific human histological findings to implant osseointegration, marginal bone stability, and long-term post-loading outcomes.

For indications prioritizing progressive graft replacement, future research should focus on biomaterials that combine initial mechanical stability with gradual replacement by vital, functional host bone. Within this therapeutic objective, the biomaterial should support cell migration, vascularization, new bone matrix formation, and subsequent remodeling [130,218]. In this context, materials with controlled resorption, optimized porosity, and surface properties that promote osteogenic cell activity appear particularly important. Selected synthetic and alloplastic biomaterials represent a promising direction, as some studies suggest that they may support favorable biological remodeling; however, their effectiveness still requires confirmation in well-designed clinical trials. Their compositional reproducibility and tunable porosity, surface properties, and resorption kinetics may also make selected synthetic scaffolds suitable platforms for controlled biological functionalization [12,35,36,85,86,144,145,146].

Future development of biologically functionalized grafts should prioritize spatially and temporally controlled delivery rather than simply increasing the amount of a biological signal [34,35,36,144]. For BMP-2, BMP-7, and PDGF, the central challenges include defining the lowest effective dose, retaining the factor within the intended compartment, matching release kinetics to the vascular and osteogenic phases of healing, and avoiding an excessive or ectopic response. For hyaluronic-acid systems and cellular concentrates such as BMAC, formulation, processing, and patient-dependent variability require greater standardization [37,38,106,107,108,109].

Recent Phase 2 evidence with expanded autologous mesenchymal stromal cells on a synthetic calcium-phosphate scaffold illustrates both the translational potential and the remaining barriers of cellular functionalization: short-term bone volume gain was encouraging, but the trial was underpowered, the required cell dose could not be produced for 7 of 36 patients allocated to the cell-therapy group (19.4%), and long-term remodeling remains unknown [215].

Future trials should compare functionalized scaffolds with otherwise identical unmodified scaffolds and should report not only ridge dimensions, but also time-dependent vital bone formation, vascularization, residual graft, integration, adverse events, and post-loading remodeling. This design would clarify whether functionalization changes the long-term biological quality of regenerated bone or merely accelerates early healing. Until such evidence is available, biologically functionalized materials should be presented as indication-specific adjuncts rather than as uniformly superior graft substitutes [37,38,107,108,109].

Overall, the main limitation of the current evidence is not only the lack of information on whether biomaterials can restore bone volume, but also the incomplete understanding of the quality and functionality of the regenerated tissue. Future research should move beyond dimensional preservation alone by jointly assessing the biological value of newly formed bone, the degree of scaffold persistence or replacement required by the indication, and long-term behavior after implant placement. Such an approach will help identify biomaterials that provide an appropriate balance between vital remodeling tissue and structural support.

## 9. Conclusions

The available evidence indicates that the evaluation of biomaterials used for bone augmentation should not be based solely on clinical defect filling or radiographic volume maintenance. Although these parameters are important for implant site development, they do not fully reflect the biological quality of the regenerated tissue. A more complete assessment should include the amount and temporal progression of newly formed vital bone, vascular ingrowth, graft integration with the surrounding host bone, graft resorption, remodeling, or persistence, and the capacity of the regenerated area to undergo subsequent physiological turnover and functional adaptation. Tissue-composition variables, including connective tissue and marrow spaces, should be interpreted within this framework.

Autogenous bone remains the biological reference material because of its osteogenic potential and ability to participate in remodeling. However, its clinical use is limited by donor-site morbidity, limited availability, and possible volumetric resorption. Xenogeneic bone substitutes, especially bovine-derived materials, are widely used and may provide favorable dimensional stability, but their slow remodeling and long-term persistence of residual particles mean that volume preservation should not be interpreted as equivalent to complete biological replacement by vital host bone. Persistent, well-integrated particles may nevertheless remain compatible with durable clinical outcomes.

Although foreign body reactions associated with residual graft particles appear uncommon and residual particles are not synonymous with treatment failure, their persistence may, in selected cases, be associated with delayed foreign body reactions, chronic inflammation, fibrotic tissue formation, or graft particle migration. These potential complications further support the need to evaluate residual biomaterial not only as a structural remnant, but also as a biologically active component of the local remodeling microenvironment. Because evidence for these events is largely case-based, it should not be used to infer that residual particles generally reduce biological quality or long-term implant success.

Allogeneic grafts and synthetic or alloplastic biomaterials represent important alternatives, although their performance depends strongly on material properties, clinical indication, and healing time. Selected modern, aseptically processed DFDBA preparations may support substantial vital bone formation and comparatively active graft replacement, but these outcomes remain product-, processing-, and formulation-dependent and should not be generalized to the entire allograft category [56]. In particular, selected synthetic and alloplastic materials, such as BCP and selected HA-based composite matrices, appear promising because they may combine structural support with progressive degradation or replacement by newly formed host bone. Nevertheless, these materials should not be treated as a homogeneous group, as their regenerative potential is strongly influenced by composition, porosity, surface characteristics, and resorption kinetics.

The main conclusion of this review is that successful bone augmentation should not be defined by augmented volume alone, but by an indication-specific balance between the formation of vital, functional host bone and the degree of scaffold persistence required for structural stability. Accordingly, biomaterial selection should be indication-specific and should balance initial space maintenance with the desired degree and timing of replacement by vital host bone. Where biological replacement is the principal objective, materials should support graft integration with host bone, functional adaptation, and physiological remodeling. In indications requiring long-term contour or space maintenance, controlled persistence of a well-integrated scaffold may also be clinically appropriate.

Biological functionalization may modify these outcomes, but current evidence supports evaluating BMP-2, BMP-7, rhPDGF-BB, hyaluronic acid, and BMAC as indication-, dose-, carrier-, and formulation-dependent adjuncts rather than as uniformly superior substitutes for an osteoconductive scaffold [37,38,106,107,108,109,110].

Future research should therefore distinguish between space maintenance and biological regeneration without assuming that one endpoint is universally superior. Long-term clinical studies combining histological, radiological, and implant-related outcomes are needed to determine how the composition of regenerated tissue influences osseointegration, marginal bone stability, and implant success. Biomaterial development should be tailored to therapeutic objectives, including either functionally relevant replacement by vital host bone or controlled persistence of an integrated scaffold when long-term structural support is required.

## Figures and Tables

**Table 2:** Summary of comparative evidence on bone remodeling and dimensional outcomes following augmentation with different grafting materials.

Study	Study Design and Comparison	Main Findings	Interpretation
**Comparisons involving multiple graft categories**			
Al-Moraissi et al. [123]	Network meta-analysis; 52 RCTs, 1483 biopsies; multiple grafts in sinus augmentation	At <6 months, autogenous bone produced 7.93 percentage points more new bone than xenografts; at ≥6 months, residual graft was 14.62 percentage points. The allograft–xenograft combination ranked highest for newly formed bone	Autogenous bone shows a more replacement-oriented remodeling profile, whereas xenografts show greater persistence. Selected graft combinations may enhance new bone formation
Khijmatgar et al. [188]	Network meta-analysis; 67 studies, 1955 patients, 2405 sinus augmentations	At residual bone height <4 mm, xenograft–allograft and xenograft–alloplast combinations and autogenous bone ranked highest; at ≥4 mm, autogenous bone ranked highest	Biomaterial performance depends strongly on local anatomical conditions and should not be interpreted independently of residual bone height
Schmitt et al. [189]	RCT; autogenous bone, allograft, BCP, and anorganic bovine bone; 5 months	New bone: 41.74 ± 2.10%, 35.41 ± 2.78%, 30.28 ± 2.16%, and 24.90 ± 5.67%, respectively. Residual graft: BCP 15.80 ± 2.10%; bovine material 21.36 ± 4.83%	Autogenous bone showed the greatest remodeling activity. Allograft and BCP supported more new bone than bovine xenograft, which showed the greatest residual persistence
**Autogenous bone versus xenogeneic substitutes**			
Correia et al. [190]	RCT; autogenous bone vs. porcine xenograft; sinus augmentation; 6 months	Total hard tissue was similar: 57.31 ± 2.91% vs. 56.01 ± 2.86%; autogenous bone was largely remodeled, whereas xenogeneic particles persisted	Comparable total hard-tissue values may conceal important differences in tissue composition and the degree of biological replacement
De Vicente et al. [191]	Clinical and histological study; autogenous bone combined with bovine HA; sinus augmentation	Newly formed bone accounted for 29%, while residual bovine material represented 21% of the augmented tissue	Even when combined with autogenous bone, xenogeneic particles may remain embedded within newly formed bone and contribute to a hybrid tissue structure
Pikdöken et al. [192]	RCT; bovine bone mineral alone vs. bovine mineral with 20% autogenous bone; 4 months	New bone: 24.19% vs. 25.73%; the difference was not significant	Adding a relatively small autogenous component did not substantially improve early bone formation and may provide limited biological benefit
Mordenfeld et al. [193]	RCT; DBBM/autogenous bone mixtures of 60:40 vs. 90:10; ridge augmentation; 7.5 months	Ridge gain: 3.5 ± 1.3 vs. 2.9 ± 1.3 mm; graft-width reduction: 37.0 ± 19.9% vs. 46.9 ± 23.5%. Histomorphometric differences were not significant	A higher autogenous-bone proportion improved dimensional outcomes, although this did not translate into significantly greater new bone formation
Pereira et al. [129]	Autogenous bone, DBBM, and their combination; sinus augmentation; 6 months	New bone in pristine, intermediate, and apical regions: autogenous bone 37.8%, 38.1%, and 44.5%; DBBM 33.4%, 32.5%, and 34.3%; combination 32.8%, 36.1%, and 27.8%	Bone formation was influenced by both graft composition and anatomical location, indicating that remodeling varies within the augmented sinus
Starch-Jensen et al. [42]	Systematic review and meta-analysis; autogenous bone alone vs. autogenous bone combined with xenograft	Volume reduction occurred with all approaches, but xenograft addition improved volumetric stability	Combining autogenous bone with a slowly resorbing xenograft improves dimensional stability, while the xenograft component may remain as a well-integrated residual scaffold
**Allogeneic grafts versus xenogeneic substitutes**			
Abushama et al. [194]	Systematic review; allografts vs. xenografts before implant placement	Both material groups were clinically effective, but substantial heterogeneity prevented demonstration of consistent superiority	Allografts and xenografts cannot be placed in a universal hierarchy because their performance depends on indication, processing, study design, and outcome measure
Kungvarnchaikul et al. [195]	RCT; DBB vs. FDBA; two-stage sinus augmentation; 6 months	FDBA showed higher RUNX2, OPN, and RANKL gene expression and greater OPN immunoreactivity	Allograft elicited a more active biological remodeling response, although the evidence was based mainly on molecular and immunohistochemical markers
Stumbras et al. [196]	RCT; bovine bone mineral vs. FDBA, both with collagen membranes; ridge preservation; 3 months	Bovine mineral showed more favorable dimensional preservation, particularly for horizontal ridge-width reduction	Greater structural persistence of xenogeneic particles may improve early dimensional preservation without necessarily indicating superior biological regeneration
Jambhekar et al. [197]	Systematic review of RCTs; xenografts vs. allografts in socket preservation	Crestal width loss: 1.30 vs. 1.63 mm; buccal-height loss: 0.57 vs. 0.58 mm. Vital bone: 35.72% vs. 29.93%; residual graft: 19.30% vs. 21.75%, respectively	Xenografts showed slightly more favorable pooled dimensional and histological values, but protocol heterogeneity limits firm conclusions
De Risi et al. [198]	Systematic review and meta-analysis; histological outcomes after ridge preservation	Highest new bone: 54.4% with allografts after 3 months; lowest: 23.6% with xenografts after 5 months. Differences among procedures were not significant	Favorable results for individual allograft protocols should not be interpreted as consistent superiority over xenogeneic materials
Foster et al. [56]	Four-arm randomized controlled human trial; particulate DFDBA, DFDBA fibers, or either form combined with xenograft; ridge preservation; 18–20 weeks	Vital bone: 37.33% and 40.76% with particulate and fiber DFDBA alone versus 24.46% and 23.85% in the respective xenograft-containing groups. Residual allograft was lower with fibers than particles, both alone (3.57% vs. 16.25%) and with xenograft (2.19% vs. 9.88%); dimensional changes did not differ significantly	Selected modern DFDBA formulations may support substantial vital bone formation and active graft turnover; physical form and processing should be reported, and similar dimensional outcomes should not be interpreted as equivalent histological regeneration
Zampara et al. [199]	RCT; 32 patients; freeze-dried cancellous allograft vs. xenograft, alloplast, and ungrafted control; ridge preservation; 3 months	Vital bone: 56.0 ± 12.8% with allograft vs. 17.2 ± 12.8% with xenograft; residual graft: 4.5 ± 7.7% vs. 22.2 ± 7.7%, respectively	The allograft showed a more replacement-oriented histological profile in this small trial, but the finding is product- and indication-specific and does not establish universal superiority
Iezzi et al. [200]; Barone et al. [201]	Human histologic cohort and related clinical case series; FDBA; ridge preservation; biopsies after 3 months; implant follow-up to 2 years	New bone: 35.22 ± 10.79%; residual graft: 12.41 ± 7.87%; bone-to-biomaterial contact: 64.61 ± 27.14%. New bone bridges surrounded residual particles without inflammatory infiltrates; all 25 implants showed 100% survival and success at 1 and 2 years	Residual allograft particles may coexist with active integration and successful clinical performance; histological composition should be interpreted together with longer-term clinical outcomes
Canullo et al. [202]	Systematic review and network meta-analysis; 88 RCTs, 2805 patients, 3073 extraction sockets	Xenografts and allografts ranked among the most predictable materials for dimensional preservation; xenografts showed a histological profile characterized by greater residual material persistence	Dimensional preservation and biological regeneration represent distinct aspects of treatment success and may not occur to the same extent
Chan et al. [203]	Systematic review; bone quality after socket preservation	Vital bone formation was heterogeneous, while residual xenogeneic particles frequently persisted and affected healed-tissue composition	Ridge dimensions or total hard tissue alone may overestimate regeneration when persistent graft particles remain within the healed site
Avila-Ortiz et al. [204]	Systematic review and meta-analysis; allografts and xenografts in ridge preservation	Both material groups reduced post-extraction ridge loss, particularly at mid-buccal and mid-lingual sites	Both groups support dimensional preservation, but these outcomes do not establish superiority in functional bone regeneration or long-term remodeling
**Alloplastic materials versus xenogeneic and other grafts**			
Tomas et al. [205]	RCT; injectable BCP vs. anorganic bovine bone; alveolar defects; 6 months	New bone: 39.91 ± 8.49% vs. 41.73 ± 13.99%; residual material: 28.61 ± 11.38% vs. 31.72 ± 15.52%; soft tissue: 31.49 ± 11.09% vs. 26.54 ± 7.25%. Differences were not significant	Injectable BCP achieved a histological outcome comparable to bovine xenograft, with slightly lower residual material persistence
Kurkcu et al. [61]	Bovine-derived HA vs. β-TCP; sinus augmentation; approximately 6.5 months	New bone: 30.13 ± 3.45% vs. 21.09 ± 2.86%; residual graft: 31.88 ± 6.05% vs. 34.05 ± 3.01%; soft tissue: 37.99 ± 5.92% vs. 44.86 ± 4.28%	A synthetic and resorbable material does not necessarily produce greater early bone formation than xenogeneic HA
Stacchi et al. [206]	Split-mouth RCT; sintered nHA vs. anorganic bovine xenograft; sinus augmentation	New bone: 34.9% vs. 38.5%; residual material: 20.6% vs. 22.3%	Both materials showed broadly comparable remodeling, with slightly more new bone for the xenograft and slightly less residual material for nHA
Tosta et al. [207]	Human histological study; synthetic substitute in sinus augmentation	Newly formed bone: 33.70%; residual graft: 32.88%; soft tissue: 33.42%	Synthetic grafts may also persist in substantial amounts and should not automatically be considered rapidly replaced by host bone
Somngam et al. [208]	Systematic review and network meta-analysis; 11 RCTs, 283 patients, 362 sinus augmentations; BCP vs. other grafts	Compared with BCP, autogenous bone and allografts produced 12.33 and 5.14 percentage points more new bone; xenografts produced 4.14 points less. Xenografts showed 6.21 points more residual material	BCP showed a replacement-oriented histological profile characterized by new bone formation and comparatively lower graft persistence; its clinical relevance remains indication-dependent
Pesce et al. [209]	Systematic review; 21 RCTs; multiple grafts in sinus augmentation	At 6 months, graft shrinkage was lowest for xenografts at 7.30 ± 15.49% and highest for autogenous grafts at 41.71 ± 12.63%	Slow xenograft remodeling represents a persistence-oriented biological endpoint that may be advantageous for long-term maintenance of augmented volume
Molly et al. [210]	Clinical study; bovine porous bone mineral, synthetic polylactic–polyglycolic acid sponge, and coral-derived calcium carbonate; extraction sockets; 6 months	Residual material: 20.2 ± 17.0%, 5.6 ± 8.9%, and 12.0 ± 16.4%, respectively	Selected synthetic materials may undergo more extensive degradation or replacement than slowly resorbing bovine mineral during socket healing
Casarez-Quintana et al. [211]	Clinical comparison; bovine-derived composite vs. HA–collagen alloplast; ridge preservation; 16 weeks	Vital bone: 26.8 ± 15.8% vs. 39.3 ± 17.8%; residual graft: approximately 18% vs. none detected. Ridge-width change at 4 mm apical to the crest: −0.9 ± 1.7 vs. −1.8 ± 1.9 mm; the dimensional difference was not significant	The alloplast supported greater vital bone formation and a more replacement-oriented histological profile, although this did not result in significantly better dimensional preservation
Canullo et al. [212]	Double-blind RCT; magnesium-enriched HA in extraction sockets; 2 vs. 4 months	Regenerated bone increased from 15.1 ± 16.7% to 77.4 ± 14.9%; residual graft decreased from 21.7 ± 15.4% to 11.6 ± 15.7%; connective tissue or marrow space decreased from 63.3 ± 22.4% to 11.0 ± 6.9%	Magnesium-enriched HA showed marked time-dependent remodeling, with substantial bone formation and decreasing residual material after 4 months
Froum et al. [213]	Pilot histological study; bioactive glass vs. DFDBA and spontaneous healing; socket preservation; 6–8 months	Vital bone: 59.5%, 34.7%, and 32.4%, respectively. Residual graft: 5.5% for bioactive glass and 13.5% for DFDBA	Bioactive glass supported high vital bone formation with limited residual material, although the evidence was based on a small pilot study
Bozza et al. [185]	Systematic review summarizing a multicenter RCT; novel bioceramic vs. bovine-derived xenograft; guided bone regeneration	Horizontal bone thickness immediately after surgery: 6.053 ± 1.262 vs. 5.907 ± 1.269 mm; after 180 ± 14 days: 5.775 ± 1.345 vs. 5.273 ± 1.285 mm	The bioceramic provided dimensional stability comparable to the bovine xenograft, although clinical evidence remains more limited than for conventional xenografts
**Biologically functionalized constructs**			
Avila-Ortiz et al. [37]; Suárez-López Del Amo and Monje [38]	Best-evidence consensus statement and systematic review; 39 articles; biologics in ridge preservation or reconstruction and implant-site development	Most clinical or radiographic outcomes were similar to conventional protocols; selected biologics improved early histomorphometry or wound healing, but superiority was not consistent in ridge or sinus augmentation	Biological adjuncts may accelerate early healing without establishing a durable advantage in long-term remodeling
Sun et al. [105]	RCT; 40 patients; rhBMP-2/biomimetic calcium phosphate (BioCaP)/β-TCP vs. β-TCP vs. spontaneous socket healing; 6 weeks	New bone: 21.18 ± 7.62%, 13.44 ± 6.03%, and 9.49 ± 0.08%; residual material: 10.04 ± 4.57% with rhBMP-2 vs. 20.60 ± 9.54% with β-TCP	Controlled rhBMP-2 delivery increased early bone formation and carrier replacement, but long-term maturation and post-loading remodeling were not assessed
Thammanichanon et al. [214]	Pilot RCT; 11 participants, 16 sockets; rhBMP-2-loaded collagen sponge vs. spontaneous healing; 12 weeks	The rhBMP-2 group showed 1.80 mm less buccal-height reduction; palatal or lingual height and overall ridge-width changes did not differ significantly	A short-term dimensional benefit does not establish improved vital bone, graft replacement, or long-term success
Jung et al. [106]; Aryal and Islam [107]	Systematic and narrative reviews; BMP-7 in regenerative dentistry; evidence largely preclinical or from early clinical applications	BMP-7 showed osteogenic potential, but controlled human evidence for alveolar augmentation and long-term outcomes remained insufficient	BMP-7 remains investigational and should not be considered interchangeable with clinically evaluated rhBMP-2 protocols
Tavelli et al. [108]	Systematic review; 63 human studies; rhPDGF-BB in oral regenerative procedures; mean follow-up 10.7 ± 3.3 months	Favorable clinical results were reported with selected allograft or xenograft combinations for GBR and ridge preservation; no serious treatment-related adverse events were identified	rhPDGF-BB may support early cellular and vascular healing, but evidence for long-term graft replacement and post-loading remodeling is limited
Lorenzi et al. [109]	Systematic review and meta-analysis; three RCTs; hyaluronic acid added to bone grafts	No statistically significant increase in new bone formation or reduction in residual graft particles was demonstrated	Improved handling or healing conditions should not be equated with proven enhancement of biological remodeling
Sauerbier et al. [110]	RCT; bovine mineral with autogenous bone vs. bovine mineral with BMAC; 3–4 months	New bone: 14.3 ± 1.8% vs. 12.6 ± 1.7%; residual bovine mineral reached 31.3 ± 2.7% in the BMAC group	BMAC did not improve early new bone formation and did not prevent substantial residual scaffold persistence
Sanz et al. [215]	Phase 2 RCT; expanded autologous mesenchymal stromal cells on a synthetic calcium-phosphate scaffold vs. autogenous bone block; 5 months	Mean between-group bone volume gain favored cell therapy by 480.01 mm^3^; implants integrated in both groups, but 7 of 36 allocated patients could not receive cells because the required yield was not achieved	Cell therapy shows translational potential, but manufacturing variability, trial limitations, and absent long-term remodeling data preclude claims of durable superiority

**Abbreviations:** BCP, biphasic calcium phosphate; BMAC, bone marrow aspirate concentrate; DBB, deproteinized bovine bone; DBBM, deproteinized bovine bone mineral; DFDBA, demineralized freeze-dried bone allograft; FDBA, freeze-dried bone allograft; HA, hydroxyapatite; nHA, nanohydroxyapatite; OPN, osteopontin; RANKL, receptor activator of nuclear factor-κB ligand; RCT, randomized controlled trial; RUNX2, runt-related transcription factor 2; β-TCP, beta-tricalcium phosphate.

**Table 3:** Comparative histomorphometric synthesis of biological properties, representative human evidence, remodeling profiles, clinical indications, and evidence quality for the major graft categories.

Graft Category and Biological Activity	Representative Human Histomorphometric Evidence (New/Vital Bone, Residual Graft, and Healing Interval)	Remodeling Characteristics and Common Clinical Indications	Quality and Limitations of Human Evidence
**Autogenous bone grafts** Osteogenic, osteoinductive, and osteoconductive; contain viable osteogenic cells and endogenous biological signals [12,19,20,21,39].	Sinus augmentation: 41.74 ± 2.10% newly formed bone after 5 months [189]. Regional new bone values of 37.8–44.5% were reported after 6 months [129]. Residual autogenous graft was not separately quantified in these comparisons; histology indicated extensive remodeling [190].	Comparatively rapid integration and graft replacement, with possible dimensional reduction. Commonly used when high biological activity or substantial horizontal or vertical reconstruction is required; donor-site morbidity and volume loss must be balanced against regenerative potential [39,40,41,42,43,49].	Moderate. Supported by randomized human trials and histology, but procedures are heterogeneous, residual autogenous graft is often not quantified separately, and long-term histological follow-up after functional loading is scarce.
**Allogeneic bone grafts** Primarily osteoconductive; selected DFDBA may show product- and processing-dependent osteoinductive activity; no viable donor osteogenic cells [11,50,51,52,53].	Mineralized cancellous allograft: 35.41 ± 2.78% newly formed bone after 5 months [189]. Selected modern aseptically processed DFDBA particles and fibers: 37.33% and 40.76% vital bone after 18–20 weeks; residual allograft 16.25% and 3.57%, respectively [56]. FDBA cohort evidence showed 35.22 ± 10.79% newly formed bone, 12.41 ± 7.87% residual graft, and 64.61 ± 27.14% bone-to-biomaterial contact after 3 months; the associated case series reported 100% implant survival and success at 1 and 2 years [200,201].	Variable, product- and processing-dependent turnover. Used in ridge preservation, guided bone regeneration, sinus augmentation, and ridge reconstruction when donor-site avoidance is desired. Selected DFDBA may support active replacement while maintaining clinically adequate ridge dimensions [56,194,196,204]. Residual FDBA particles may coexist with new-bone bridging, absence of inflammatory infiltrates, and successful implant performance [200,201].	Limited to moderate. Randomized human histological studies are available, including product-specific DFDBA trials, but findings depend strongly on processing and physical form, and direct long-term histology-clinical correlations remain limited. Recent cohort evidence provides a direct, although still limited, link between human histology and 2-year implant outcomes.
**Xenogeneic bone grafts** Predominantly osteoconductive; support cellular and vascular ingrowth; minimal intrinsic osteoinductive activity and no viable osteogenic cells [5,12].	Anorganic bovine bone: 24.90 ± 5.67% newly formed bone and 21.36 ± 4.83% residual graft after 5 months [189]. In pooled ridge-preservation data, mean vital bone and residual graft were 35.72% and 19.30%, respectively [197].	Slow or incomplete resorption with persistent but often integrated particles and favorable dimensional stability. Commonly used in sinus augmentation, ridge preservation, and contour or space maintenance; persistence should not be equated with treatment failure [5,11,25,42,75,76,196,204].	Moderate to comparatively extensive. Multiple randomized trials, systematic reviews, human histology, and long clinical follow-up are available; however, the relationship between residual particles, rare foreign body events, and long-term implant outcomes remains uncertain.
**Alloplastic biomaterials** Primarily osteoconductive; generally lack viable cells and intrinsic osteoinductive activity; resorption is formulation-dependent [83,84,85,86].	BCP: 30.28 ± 2.16% newly formed bone and 15.80 ± 2.10% residual graft after 5 months [189]. Injectable BCP: 39.91 ± 8.49% newly formed bone and 28.61 ± 11.38% residual material after 6 months [205]. HA–collagen alloplast: 39.3 ± 17.8% vital bone with no detectable residual graft after 16 weeks [211].	Highly formulation-dependent: β-TCP and selected composites may undergo progressive replacement, whereas HA-rich materials may persist longer. Used in ridge preservation, sinus augmentation, contained defects, and graft combinations when standardized availability and tunable handling or resorption are priorities [5,12,78,205,206,207,208,209,210,211,212,213].	Limited to moderate and formulation-specific. Human trials exist for selected BCP, HA, and composite products, but marked material heterogeneity and scarce long-term post-loading histology preclude generalization to the entire alloplastic category.

**Note:** Detailed information on graft origin, representative forms, general biological properties, advantages, and limitations is provided in Table 1, while individual comparative studies are summarized in Table 2. Histomorphometric terminology is retained as reported by each study; “newly formed bone” and “vital bone” should not be assumed to be interchangeable. Percentages are representative study-specific findings rather than pooled estimates for entire graft categories and should be interpreted in relation to the specific material, clinical procedure, anatomical site, and healing interval. Evidence quality is a qualitative appraisal based on study design, consistency, product specificity, and the availability of long-term human data rather than a formal GRADE assessment. **Abbreviations:** BCP, biphasic calcium phosphate; DFDBA, demineralized freeze-dried bone allograft; GBR, guided bone regeneration; HA, hydroxyapatite; β-TCP, beta-tricalcium phosphate.

## Data Availability

No new data were created or analyzed in this study. Data sharing is not applicable to this article.

## Abbreviations

The following abbreviations are used in this manuscript:

BCP	biphasic calcium phosphate	
BMAC	bone marrow aspirate concentrate	
BMP	bone morphogenetic protein	
CBCT	cone-beam computed tomography	
CT	computed tomography	
DFDBA	demineralized freeze-dried bone allograft	
DBB	deproteinized bovine bone	
DBBM	deproteinized bovine bone mineral	
FDBA	freeze-dried bone allograft	
GBR	guided bone regeneration	
HA	hydroxyapatite	
micro-CT	micro-computed tomography	
nHA	nanohydroxyapatite	
NMA	network meta-analysis	
OPN	osteopontin	
PDGF	platelet-derived growth factor	
RANKL	receptor activator of nuclear factor-κB ligand	
RCT	randomized controlled trial	
rhBMP-2	recombinant human bone morphogenetic protein 2	
rhPDGF-BB	recombinant human platelet-derived growth factor BB	
RUNX2	runt-related transcription factor 2	
β-TCP	beta-tricalcium phosphate	
